# Replisome Proximal Protein Associations and Dynamic Proteomic Changes at Stalled Replication Forks

**DOI:** 10.1016/j.mcpro.2024.100767

**Published:** 2024-04-13

**Authors:** Carla-Marie Jurkovic, Jennifer Raisch, Stephanie Tran, Hoang Dong Nguyen, Dominique Lévesque, Michelle S. Scott, Eric I. Campos, François-Michel Boisvert

**Affiliations:** 1 Faculty of Medicine and Health Sciences, Department of Immunology and Cell Biology, Université de Sherbrooke, Sherbrooke, Québec, Canada; 2 Genetics & Genome Biology Program, Department of Molecular Biology, The Hospital for Sick Children, University of Toronto, Toronto, Ontario, Canada; 3 Faculty of Medicine and Health Sciences, Department of Biochemistry and Functional Genomics, Université de Sherbrooke, Sherbrooke, Québec, Canada

**Keywords:** replication fork, DNA replication, DNA repair, proteomics, interactome, hydroxyurea, BioID, biotinylation

## Abstract

DNA replication is a fundamental cellular process that ensures the transfer of genetic information during cell division. Genome duplication takes place in S phase and requires a dynamic and highly coordinated recruitment of multiple proteins at replication forks. Various genotoxic stressors lead to fork instability and collapse, hence the need for DNA repair pathways. By identifying the multitude of protein interactions implicated in those events, we can better grasp the complex and dynamic molecular mechanisms that facilitate DNA replication and repair. Proximity-dependent biotin identification was used to identify associations with 17 proteins within four core replication components, namely the CDC45/MCM2-7/GINS helicase that unwinds DNA, the DNA polymerases, replication protein A subunits, and histone chaperones needed to disassemble and reassemble chromatin. We further investigated the impact of genotoxic stress on these interactions. This analysis revealed a vast proximity association network with 108 nuclear proteins further modulated in the presence of hydroxyurea; 45 being enriched and 63 depleted. Interestingly, hydroxyurea treatment also caused a redistribution of associations with 11 interactors, meaning that the replisome is dynamically reorganized when stressed. The analysis identified several poorly characterized proteins, thereby uncovering new putative players in the cellular response to DNA replication arrest. It also provides a new comprehensive proteomic framework to understand how cells respond to obstacles during DNA replication.

## Highlights

• Protein associations for 17 replication fork components are provided as a resource.
• Identification of proteomic changes due to hydroxyurea-induced replicative stress.
• Molecular insights on fork dynamics and DNA replication repair modulators.

## Graphical Abstract

**Figure:**
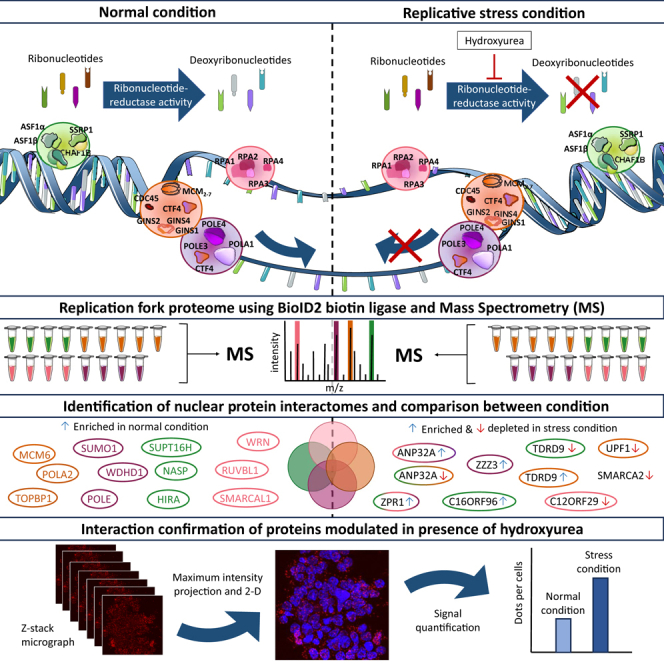

## In Brief

DNA replication repair was examined by comparing proximal protein associations for 17 core components of the replication fork in cycling cells and cells facing genotoxic stress. An important reorganization of the interactome was observed upon a hydroxyurea treatment, with 108 associations being either markedly lost or gained. This high-resolution analysis informs on replication fork protein complexes, organization, and stress responses. Our rich data resource includes interactome data for components of the CDC45/MCM2-7/GINS (CMG) helicase, histone chaperones, and DNA polymerases.

DNA replication occurs in three steps: initiation, elongation, and termination, completed once per cell cycle. Local unwinding of DNA at origins of replication in S phase gives rise to replication bubbles with bidirectional DNA replication. Replication forks at either end of the bubble comprise an unwinding parental DNA strand and two replicating sDNA templates (*i.e.*, the leading and lagging strands). Genotoxic stressors cause replication fork stalling. There are therefore repair mechanisms to ensure that DNA is faithfully replicated. This requires a close coordination between the replication machinery and repair factors, which is important for replication fork progression. Failure to promptly repair replicating DNA results in potentially serious replication errors and genomic instability.

**Fig. 1:**
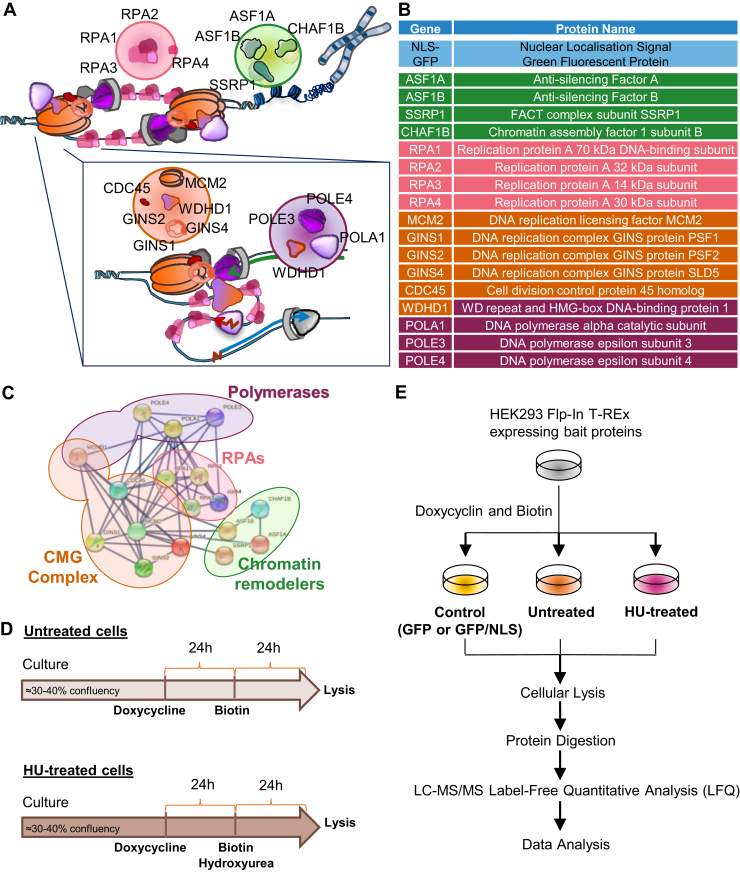
**BioID of proteins within four functional categories, needed for DNA replication.***A*, *schematic representation* of the replication fork and the 17 bait proteins analyzed by BioID. *B*, functional grouping of the BioID bait proteins. *Purple, orange, pink, and green*, respectively, represent DNA polymerases and associated factors, CMG helicase, RPA proteins, and histone chaperones. *C*, STRING network of the 17 bait proteins grouped by functional cluster. *Lines* represent physical associations with the highest confidence scores. *D* and *E*, experimental pipeline. Bait protein expression was induced, cells were exposed to biotin and hydroxyurea when due, and lysed. Biotinylated proteins were affinity purified, trypsinized, and analyzed by mass spectrometry. BioID, biotin identification; CMG, CDC45/MCM2-7/GINS; RPA, replication protein A.

The initiation step can be divided into two stages. The first involves the assembly of prereplicative complexes at origins of replication at the end of mitosis, that is, when loading of the MCM2-7 subunits of the replicative helicase occurs. Two hexameric MCM2-7 rings are loaded at origins of replication in a head-to-head configuration. This is followed by the assembly of a preinitiation complex at the end of the G1 phase of the cell cycle (1, 2), where CDC45/MCM2-7/GINS (CMG) helicase holoenzymes form. The second event occurs at the onset of S phase and involves MCM protein phosphorylation and the firing of origins of replication (3). The CMG helicases at origins of replication then start unwinding DNA in a bidirectional manner, giving rise to a pair of DNA replication forks within each replication bubble (Fig. 1*A*). The exposed ssDNA is stabilized by the replication protein A (RPA) complex to protect it from endonucleases and prevent aberrant reannealing. The trimeric RPA1-3 complex has additional functions, including the recruitment and stabilization of DNA repair complexes (4). Nucleosomes restrict DNA accessibility and thus represent an obstacle for the CMG helicase during the elongation stage of DNA replication. Mechanisms exist to evict histones immediately ahead of replication forks and to reassemble nucleosomes on the nascent DNA strands (5). This requires several histone chaperones, whose activities at the replication fork appear to be largely preserved amongst eukaryotes. FACT and the MCM2 subunit of the CMG helicase both possess histone chaperone activity and are minimally needed to cooperatively disassemble nucleosomes in front of the replication fork (6). WDHD1 (CTF4/AND-1) and polymerase alpha (Pol α) redeposit the recycled histones on the lagging strand (though the bulk of the lagging strand replication is done by polymerase δ), while the POLE3/4 subunits of Pol ε recycle histones on the leading strand (7, 8, 9). While the anti-silencing factor 1 (ASF1) histone chaperone can bind evicted histones, it also transfers new histones to chromatin assembly factor 1 (CAF-1) to fully restore nucleosome density on nascent DNA (10, 11, 12).

DNA replication thus requires the mobilization of multiple proteins, which together form the replisome. The concerted activity of the enzymes needed to unwind and duplicate DNA (*e.g.*, helicases, primases, and polymerases) is further supported by a multitude of factors that solve challenges inherent to DNA replication. Proteins within the replisome preserve genetic information and have high essentiality scores (13). In addition to replicating DNA, the replisome interacts with a multitude of DNA repair factors needed to promptly respond to disturbances such as replicative stress and to preserve genome stability. The improper recruitment of proteins threatens the replication fork and can result in its collapse and shutdown. Hydroxyurea (HU) depletes deoxynucleotide pools, thereby slowing replication and arresting cells at the G1/S border (14). Prolonged HU treatments induce replication stress and DNA damage (15). Such damage can be bypassed by leading strand repriming but the process is inefficient (15).

Proximity-dependent biotin identification using BioID2 (16) coupled with mass spectrometry (MS) was used to quantitatively identify proteins in close proximity to 17 core replisome components. That allowed us to build a comprehensive interactome database for bait proteins that were either components of the CMG replicative helicase, the RPA complex, DNA polymerases, or histone chaperones with central roles at replication forks. Because the interactions are expected to change upon replication stress, we again repeated the analysis in HU-treated cells. In all, the analysis revealed a vast proximity association network involving 654 proteins, of which 108 responded to HU. The many reciprocal BioID2 analyses herein recapitulated key results and informed on the spatial organization of proteins at the replisome. The prey proteins identified here include factors whose precise cellular function(s) remain poorly characterized, opening the door to future exploration.

## Experimental Procedures

### Generation of Inducible Stable Cell Lines and HU Treatment

Oligonucleotides-containing *attB* sites were used to amplify ASF1A, ASF1B, CDC45, CHAF1B, MCM2, GINS1, GINS2, GINS4, POLA1, POLE3, POLE4, SSRP1, and WDHD1 complementary DNA (cDNAs) from an U2OS (ATCC HTB-96) cDNA library by PCR with the polymerase TransStart KD Plus (Transgenbiotech #AP301). The cloning of RPA1-4 was recently described by us elsewhere (17).

AttB recombination using BP clonase II (Life Technologies) was carried out between the PCR products and the *attP* acceptor-containing pDONR221 plasmid (Life Technologies). The BP reaction products were transformed into DH10β bacteria and selected using kanamycin. Once sequenced, the validated ASF1A/B, RPA1 to 4 clones were used for AttR recombination *via* LR clonase II TM (Life Technologies) with the pgLAP1-3MYC-BioID2, a modified version pgLAP1 (Addgene #19702), while validated CDC45, CHAF1B, MCM2, GINS1, 2, 4, POLA1, POLE3/4, SSRP1, and WDHD1 cDNA was introduced into pDEST-pcDNA5-FLAG-BioID2 (kind gift from Dr Anne-Claude Gingras). All constructs encoded the bait proteins with an N-terminal BioID2 enzyme. LR reaction products were transformed into DH10β bacteria and selected using ampicillin.

Control plasmids encoding GFP and GFP-nuclear localization sequence (NLS) BioID2 fusion proteins were also prepared. Isogenic stable HEK293 Flp-In T-REx (293FT) cells (Thermo Fischer Scientific #R78007) were obtained by cotransfecting each BioID2 construct along with the pOG44 plasmid (Life Technologies). The transfected cells were selected by adding 100 μg/ml hygromycin B (Wisent) and 100 μg/ml blasticidin-HCl (Wisent) to the culture media.

### Cell Culture

293FTs were cultured as adherent cells in Dulbecco's Modified Eagle Medium containing 4.5 g/l glucose, L-glutamine, and sodium pyruvate (Wisent), supplemented with 10% FB essence, 20 IU/ml penicillin/20 μg/ml streptomycin, and 2.5 μg/ml amphotericin B (#540-201-EL and 450-105-QL; Wisent). Hygromycin B and blasticidin were again included in the cultures. Protein expression was induced with 2.5 μg/ml doxycycline (Clontech Laboratories) for 48 h and, in case of DNA damage condition, cells were treated for the last 24 h with 1 mM HU (EDM Milipore; #40046-5GM).

### Immunoblotting

Cells were washed with 1× PBS, lysed in 1× Laemmli, and sonicated 2× 10s on ice at an intensity of 25% (Fisher Scientific Sonicator #FB120110). The protein concentrations were quantified using a bicinchoninic acid assay, and 25 μg of extract was resolved by SDS-PAGE for each sample. The proteins were transferred to a nitrocellulose membrane (Bio-Rad; #1620115) using Bjerrum Schafer-Nielsen transfer buffer containing 20% ethanol. Proteins were detected using antibodies against MYC at 1:50 (ATCC, #CRL-1729), FLAG at 1:1000 (Sigma, #F1804), γH2AX at 1:1000 (Cell Signaling, #97185), β-tubulin at 1:1000 (9F3, Cell Signaling, #2128S), or GAPDH at 1:50,000 (Cell Signaling, #5174S). Anti-rabbit horseradish peroxidase (Cell Signaling, #7074S) and anti-mouse horseradish peroxidase (Cell Signaling, #2128S) secondary antibodies were used at 1:1000. Signals were obtained using the Clarity Western enhanced chemiluminescent Substrate kit (Bio-Rad, #1705061) and imaged on a Chemidoc XRS using the ImageLab software (Bio-Rad; https://www.bio-rad.com/en-ca/product/image-lab-software?ID=KRE6P5E8Z).

### Proximity Ligation Assay

Experiments were performed using a proximity ligation assay (PLA) Kit (DUO92101-1 KT; Sigma-Aldrich). Cells were seeded onto coverslips in 12-well plates, fixed in 4% paraformaldehyde (Electron Microscopy Sciences, #15713) for 20 min at 4 °C, and washed twice with 1× PBS. Cells were then permeabilized with 0.25% Triton X-100 for 5 min at 4 °C and washed twice with 1× PBS. Samples were saturated with the kit’s blocking solution for 1 h at 37 °C, after labeling, and proceeded as prescribed by the manufacturer, using different antibody pairs (Myc 1:2, ATCC #CRL-1729GR; FLAG 1:500, Sigma #F1804; ANP32A 1:200, ABclonal #A5768; ZPR1 1:200, ABclonal #A15746). The samples were mounted using Duolink *in situ* mounting medium containing 4′,6-diamidino-2-phenylindole and visualized using a Zeiss LSM 880 two-photon confocal microscope and the Zen lite Black software. Images were analyzed using the Zen lite Blue software (https://www.zeiss.com/microscopy/en/products/software/zeiss-zen-lite.html) and the number of signals per cell was quantified from as many cells as possible using ImageJ (https://imagej.net/ij/download.html).

### Immunolabeling and Microscopy

293FT cells were grown on round glass coverslips to ∼60% confluency. A slightly different protocol was used for FLAG- and MYC-tagged proteins. All washes were in 1× PBS. For FLAG immunolabeling, the coverslips were washed and fixed with a solution of 4% paraformaldehyde and 4% sucrose for 15 min at room temperature. Cells were washed twice for 5 min and permeabilized with 0.25% Triton X-100 for 5 min at 4 °C. Samples were washed again and blocked with 10% bovine serum albumin (BSA) for 30 min at 37 °C in a humid chamber. Cells were then incubated with the FLAG antibody overnight at 4 °C in the dark (Sigma #F1804, diluted at 1:1000 in 3% BSA solution). For MYC immunolabeling, the coverslips were washed and fixed with 4% paraformaldehyde for 20 min at 4 °C. Cells were then washed, permeabilized with 0.15% Triton X-100 for 5 min at 4 °C, and washed once more. Samples were blocked with a 10% goat serum solution for 20 min at 4 °C and incubated overnight at 4 °C with the MYC antibody at 1:10 (ATCC, #CRL-1729). All samples were then washed and incubated with Alexa Fluor anti-mouse 488 secondary antibody (Invitrogen, #A11001) at a 1:800 dilution for 1 h at room temperature.

For colocalization assays at active replication foci, 293FTs expressing the proteins of interest were grown on glass coverslips coated with poly-L-lysine (Sigma, #P4707) and induced with doxycycline for 48 h at ∼ 60% confluency, following a 2-h with 2 mM HU to accumulate cells in S phase. Cells were washed twice and fixed for 15 min at room temperature with 4% paraformaldehyde diluted in 1× PBS. After three 5 min washes, cells were permeabilized with cold methanol for 10 min at −20 °C. Cells were washed once more and incubated with a blocking solution (5% normal goat serum, 0.3% Triton X-100 in 1× PBS) for 1 h at room temperature. We then incubated cells with primary antibodies diluted in antibody dilution buffer (1× PBS, 1% BSA, 0.3% Triton X-100) namely, FLAG (Cell Signaling #14793S, 1:400), proliferating cell nuclear antigen (PCNA) (Cell Signaling #2586S, 1:1000), and Myc (Cell Signaling #2278, 1:400). Coverslips were washed and incubated with Alexa Fluor anti-mouse 488 (Invitrogen, #A11001) or Alexa Fluor anti-rabbit 546 (Invitrogen, #A11010) secondary antibodies at a 1:800 dilution for 1 h at room temperature. Cells were finally stained with 1 μg/μl 4′,6-diamidino-2-phenylindole for 5 min (Biotium, 40,011) and slides were assembled using Immu-mount mounting medium (Thermo Fisher Scientific; #9990402). Images were acquired using a Zeiss LSM 880 two-photon confocal microscope, and the images were analyzed using the Zen lite Black software (Zeiss).

### MS Protein Interaction Studies

#### Purification of Biotinylated Proteins

For each BioID bait protein, a 100 mm Petri dish with the corresponding cell line was cultured to 40 to 50% confluency. Protein expression was induced by adding 2.5 μg/ml doxycycline (Clontech Laboratories) for 48 h; and 50 μM biotin (Sigma-Aldrich) and 1 mM HU (when applicable; EDM Millipore; 40046-5 GM) were added to the medium during the last 24 h of induction. Cells were harvested by trypsinization and collected by centrifugation at 1500*g* for 5 min at 4 °C.

Cell pellets were resuspended in freshly prepared lysis buffer containing 8 M urea, 50 mM Hepes pH 7.4, 1 mM PMSF, and 1 mM DTT. The samples were then transferred to protein low bind tubes and sonicated twice for 10 s on ice at an intensity of 30% (Fisher Scientific Sonicator #FB120110). Samples were clarified by centrifugation at 16,500*g* for 10 min at 4 °C and mixed with 50 μl of a slurry of prewashed high-performance streptavidin sepharose beads (Cytiva; #17511301) in 1 ml lysis buffer. The tubes were placed on a rotating wheel at 4 °C overnight and centrifuged at 1000*g* for 5 min at 4 °C. The supernatant was carefully removed and beads were washed five times with a buffer composed of 8 M urea and 50 mM Hepes pH 7.4. The final bead suspension was transferred to fresh low bind tubes prior to the tryptic digestion.

#### Tryptic Digestion

All buffers listed below were prepared using MS-grade water (Thermo Fisher Scientific). The sepharose beads were washed four times with 20 mM ammonium carbonate, followed by a final wash with 20 mM ammonium bicarbonate and 1 mM biotin to saturate all of the streptavidin on the beads. Proteins were reduced by incubating the beads in 50 μl of 20 mM ammonium bicarbonate and 10 mM DTT for 30 min at 60 °C with stirring at 1250 rpm. Alkylation was carried out by adding 50 μl of 20 mM ammonium bicarbonate and 15 mM chloroacetamide to the samples and incubating for 1 h at room temperature in the dark while shaking. Chloroacetamide was quenched by adding 15 mM DTT, and beads were finally incubated overnight at 37 °C with 1 μg MS-grade trypsin at a final concentration of 10 μg/ml (Thermo Fisher Scientific, #PI90058). The samples were acidified by adding formic acid (FA) to a final concentration of 1%, and beads were centrifuged at 2000*g* for 3 min before collecting the supernatants and transferring them to new low bind tubes. Beads were finally resuspended in 100 μl of a 60% acetonitrile (ACN) (Sigma-Aldrich) and 0.1% FA buffer and stirred at 1250 rpm for 5 min at room temperature. The supernatant was carefully collected and combined with the first one. Samples were concentrated using a centrifugal evaporator at 60 °C until completely dry and resuspended in 30 μl 0.1% TFA (Sigma-Aldrich).

Peptides were desalted using 10 μl ZipTip micropipette tips containing a C18 resin (EMD Millipore). The ZipTips were first humidified by aspirating 10 μl of 100% ACN three times and equilibrated by aspirating 10 μl of 0.1% TFA three times. Ten microliters of each sample was pipetted ten successive times in its respective ZipTip column. This step was repeated three times to pass the entire samples through the ZipTips. The ZipTips were then washed three times with 10 μl of 0.1% TFA. Peptides were eluted into fresh low-binding microtubes by again aspirating 10 μl of 50% ACN and 1% FA ten times. The step was carried out three times to obtain 30 μl per sample. The peptides were concentrated using a centrifugal evaporator at 60 °C until completely dry and resuspended in 30 μl of 1% FA buffer. Peptide samples were quantified using a NanoDrop spectrophotometer (Thermo Fisher Scientific), transferred to glass vials (Thermo Fisher Scientific), and stored at −20 °C until their analysis by MS.

#### MS Interactome Analysis

For LC-MS/MS, 250 ng of each sample was injected into an HPLC system (nanoElute, Bruker Daltonics), loaded onto a trap column at a constant flow rate of 4 μl/min (Acclaim PepMap100 C18, 0, 0, 3 mm id × 5 mm, Dionex Corporation), and eluted onto a C18 analytical column (bead size 1.9 μm, 75 μm × 25 cm, PepSep). Peptides were finally eluted from the last column through an ACN gradient (5–37%) carried over 2 h in 0.1% FA at 500 nl/min while being further injected into a timsTOF Pro ion mobility mass spectrometer equipped with a source of electrospray nano Captive Spray (Bruker Daltonics). Data were acquired using data-dependent auto-tandem mass spectrometry with a mass range of 100 to 1700 *m/z*, with parallel accumulation–serial fragmentation enabled and the number of parallel accumulation–serial fragmentation analyses set at ten duty cycles of 1.27 s and a dynamic exclusion of 0.4 min, isolation window dependent on *m/z*, and collision energy of 42.0 eV. The target intensity was set at 20,000, with an intensity threshold of 2500.

#### Identification of Proteins *via* MaxQuant

Raw files were analyzed using the MaxQuant software version 1.6.17.0 (https://www.maxquant.org/) and the UniProt human proteome database (03/21/2020, 75,776 entries). The parameters used for the MaxQuant analysis (with the trapped ion mobility spectrometry - data-dependent acquisition type in the group-specific parameters) were as follows: two cleavage errors were allowed; the fixed modification was carbamidomethylation on cysteine; the enzyme was trypsin (K/R not before P); variable changes included in the analysis were methionine oxidation, N-terminal protein acetylation, and protein carbamylation (K, N-terminal). A mass tolerance of 10 ppm was used for precursor ions and a tolerance of 20 ppm was used for fragment ions. The identification values “peptide-spectrum match false discovery rate (FDR),” “protein FDR,” and “site decoy fraction” were set to 0.05. The minimum number of peptides was set to 1. Label-free quantification was also selected with a minimum number of label-free quantification ratios of 2. The “Second peptides” and “match between series” options were also allowed.

#### Experimental Design and Statistical Analysis

Biological triplicates were analyzed for each of the 17 protein baits and the MS again included two further technical replicates to properly assess variability and technical errors. The data from all replicates was analyzed using proteomic statistical analysis with R (18) with the following parameters: filtering out reverse, contaminants, only identified by site, and proteins identified with a minimum of three unique peptides. The treatment of missing values corresponding to a maximum of two missing values for partially observed conditions (termed partially observable value authorized per condition only while removal of values missing on the entire condition where removed for both conditions. Finally, median normalization; SLSA imputation on partially observable values and DetQuantile (1% with 0.2 factor) on missingvalues on the entire conditions were applied. The data was normalized by mean centering within conditions, and the results were sorted to select proteins identified in at least two of the three biological replicas.

Data obtained from untreated cells was analyzed using SAINTexpress (version 3.6.1; https://saint-apms.sourceforge.net/Main.html) (19) to score the probability that prey proteins identified by our baits were also significantly enriched above background levels (from a negative control) using a Bayesian statistical model and MS/MS counts. Each bait protein was compared to three negative controls namely, HEK293 Flp-In T-REx (293FT) cells not expressing any bait protein, cells expressing BioID2-GFP, and cells expressing BioID2-GFP-NLS. Prey proteins were considered actual proximal associations when the SAINT score was ≥0.95 (using the 293FT control) and AvgSpec ≥5. These proteins were frequently found to have a FoldChange ≥1.5 over the GFP-BioID2 or GFP-NLS-BioID2 controls (supplemental Tables S1–S17). The network analyses with functional enrichment only considered nuclear proteins and the dot plots were generated using Cytoscape software version 3.8.2 (https://cytoscape.org/), its ClueGO version 2.5.7 extension, and ProHitz-*viz.* (20, 21, 22).

Results obtained using HU-treated cells were also sorted using proteomic statistical analysis with R as explained above. After mean centering within each condition, the results were sorted to again select proteins present in at least two of the three biological replicates (supplemental Tables S1–S17). The average intensity values were obtained using the three biological replicates. A binary logarithm was applied to the means to calculate the enrichment ratio: bait (± HU)/(untreated GFP-NLS). Scatter plots comparing the ratios obtained in normal conditions and with DNA damage were generated, and enrichment rates were considered significant if log2(Fold Change) ≥1 (supplemental Figs. S8, S10, S12, and S14). Proteins significantly more abundant in one condition than the other were filtered out based on their intensity values greater than the interquartile threshold specific to each distribution (supplemental Figs. S9, S11, S13, and S15). The enrichment ratios for each quantified protein were compared across isoforms using the BioVenn and DeepVenn interfaces (23). Cytoscape software version 3.8.2 (21), its ClueGO (22) version 2.5.7 extension, and ProHitz-*viz.* (20) were used to obtain the enriched function arrays for each protein of interest and their comparison in both conditions.

## Results

### Using BioID to Interrogate the Replication Fork Proteome

Proximity-dependent BioID was used to identify proteins found in the vicinity of replication forks in normal circumstances or upon stress. Bait proteins were fused to the BioID2 enzyme to tag proteins within a ∼10 nm radius (16). The approach is advantageous over affinity purifications, where harsh solubilization steps risk disrupting chromatin-bound protein complexes. A total of 13 proteins with roles in DNA replication were selected (Fig. 1), in addition to four RPA proteins recently analyzed by us (17). These 17 proteins encompassed four functional clusters namely, the replicative CMG helicase complex, RPA complexes, DNA polymerases ε (leading strand) and α (lagging strand), and histone chaperones needed for replication on chromatin (Fig. 1, *A* and *B*).

#### CMG Complex

The CMG helicase unwinds the DNA double helix and spearheads replication forks (24, 25). We selected the MCM2 component of the hexameric MCM2-7 ring given that it has a dual function as a motor subunit of the CMG helicase and as a histone chaperone that evicts histones ahead of the fork (26). CDC45 and the GINS1, GINS2, and GINS4 subunits of the tetrameric GINS subcomplex were also included as activating subunits of the CMG complex (GINS3 and consequences of GINS3 mutations were previously shown by us (27)) (24, 28).

#### Polymerases

We further include WDHD1, which links the CMG helicase and Pol α on chromatin (29, 30), and the POLA1 catalytic subunit of Pol α. These proteins enable Okazaki fragments synthesis by DNA polymerase δ and confer stability (31, 32). Included in the proteomic analysis were also the POLE3/4 subunits of DNA polymerase ϵ that synthesizes the leading strand (33, 34). POLE3/4 subunits have histone chaperone activity and enable histone redeposition on the leading strand, while WDHD1 and Pol α proteins similarly facilitate histone recycling on the lagging strand (8, 9). This process is crucial for maintaining chromatin integrity during DNA replication and for the recycling of evicted histones within the replisome.

#### RPA Complex

The RPA1, RPA2, and RPA3 proteins form a heterotrimeric complex that binds and protects ssDNA generated by CMG helicase movement during DNA replication but also through the process of DNA recombination and repair (4). RPA also serves as a recruitment platform for proteins involved in fork stabilization. RPA4 can replace RPA2 during DNA repair to form the alternative RPA complex (35, 36). This complex can oppose the functions of canonical RPA, at least in the context of disease-causing somatic instability (17). We here leverage the proteomes of RPA1 to 4, recently obtained by us through that study.

#### Histone Chaperones

The FACT histone chaperone and the MCM2 subunit of the CMG helicase cooperatively disassemble nucleosomes ahead of the replication fork. Along with ASF1, they further chaperone the evicted histones for recycling behind the replication fork (6, 26, 37). ASF1 also works in conjunction with CAF-1 to assemble nucleosomes using new histones during replication or DNA repair (38, 39, 40). We therefore chose to follow these proteins using the p60 (CHAF1B) subunit of CAF-1 and the SSRP1 subunit of FACT the complex. FACT is involved in several chromatin-related processes, including DNA replication, repair, and transcription (41, 42, 43, 44).

Established interactions amongst those proteins are shown in Figure 1*C*. The STRING (45) network highlights functional and physical protein associations, backed by high confidence scores (≥0.900). The following sections explore how the proximal associations enriched this current knowledge, taking functional overlaps (such as that of WDHD1 that links Pol α to the CMG helicase) into consideration.

### Expression and Localization of Bait Proteins With and Without HU

Isogenic HEK293 Flp-In T-REx (293FT) cells expressing one of the 17 BioID2 fusion proteins were induced and supplemented with biotin for 48 h. HU was added during the last 24 h when probing the proteomes in cells with arrested replication forks (Fig. 1*D*). Parental 293FT and cells expressing BioID2 fused to GFP or GFP-NLS were used as controls for nonspecific nuclear background labeling (Fig. 1*E*).

**Fig. 2:**
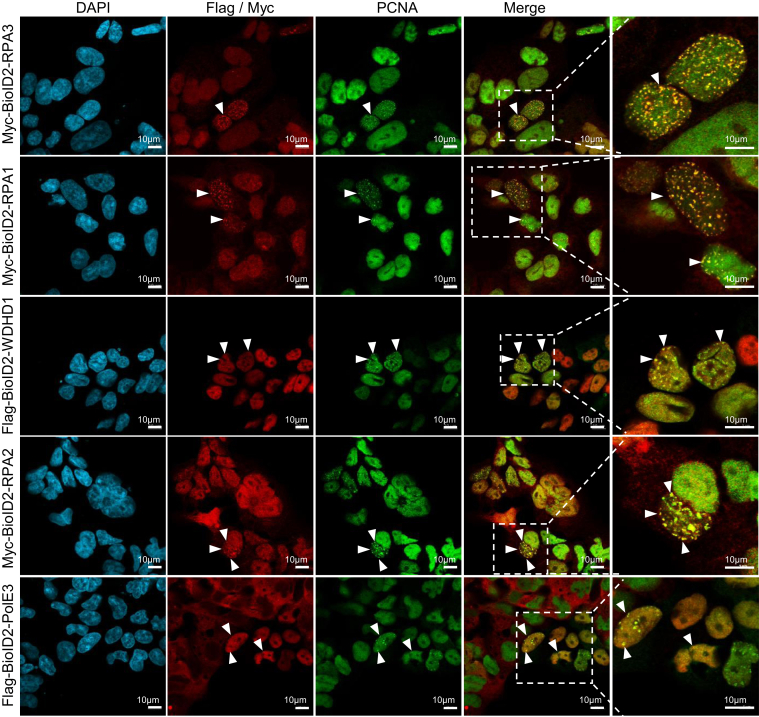
**Immunofluorescent labeling showingproliferating cell nuclear antigencolocalization with the epitope-tagged bait proteins.** Cells were induced with doxycycline for 48 h and exposed to 2 mM HU for 2 h to enrich for cells in S phase. The scale bars represent 10 μm. HU, hydroxyurea.

DNA damage induction in 293FT was assessed by exposing cells to increasing HU concentrations for 24 h and using γH2AX as a proxy for damage in immunoblots and immunofluorescence (supplemental Fig. S1, *A* and *B*). A 1 mM HU concentration was thereby chosen for our subsequent experiments. The same concentration is known to induce DNA replication stalling (46), and our flow cytometry analysis confirmed an accumulation of HU-treated cells at the G1/S border (supplemental Fig. S1, *C* and *D*). Expression of the BioID2 fusion proteins was then verified with and without HU. The stable 293FT lines were induced with doxycycline for 24 h and protein expression and subcellular localizations were assessed by immunoblotting and immunofluorescence microscopy, respectively. All 17 bait proteins were expressed at the expected molecular weight (supplemental Fig. S1, *E* and *U*), and signals for the proteins were overwhelmingly nuclear (Fig. 2 and supplemental Fig. S2), consistent with their expected endogenous localization (47). Moreover, to validate an association of our bait proteins with DNA replication foci, we evaluated their colocalization with proliferating cell nuclear antigen (PCNA) by immunofluorescence. As exemplified by RPA1, RPA2, RPA3, WDHD1, and POLE3 in Figure 2, there was clear colocalization of the bait proteins with PCNA after a 2 h low-dose HU treatment (used here to enrich for cells in S phase).

### Proteomes Within the Replication Fork

**Fig. 3:**
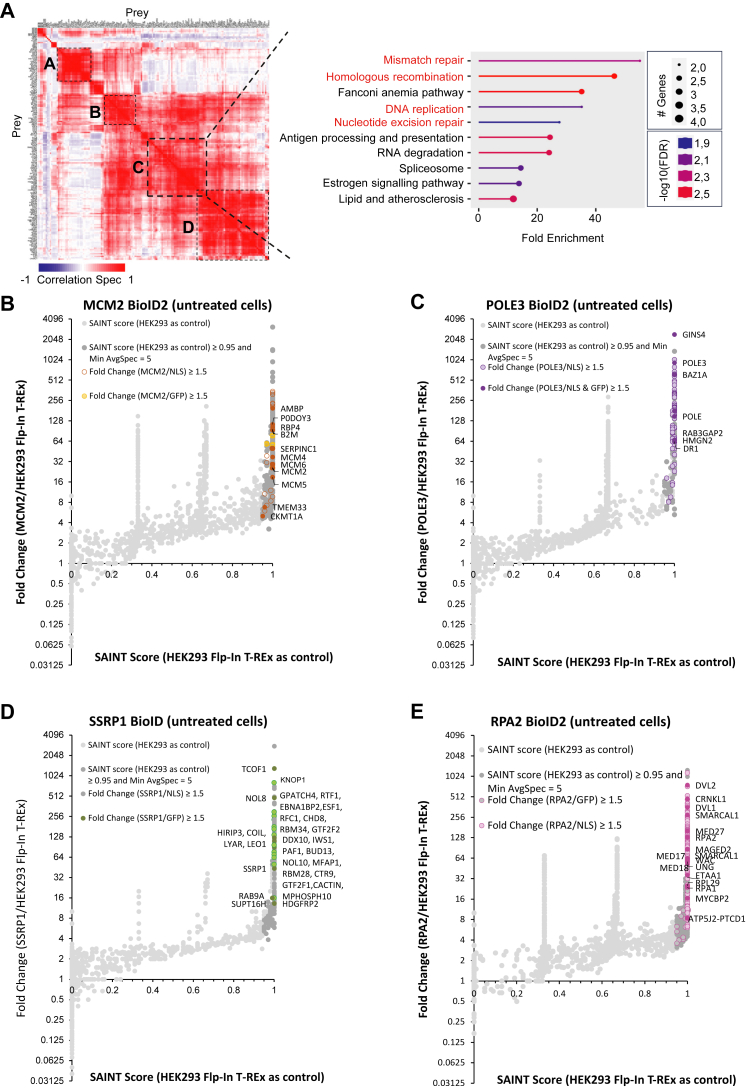
**SAINT analysis and functional clustering of prey proteins.***A*, heatmap depicting Pearson’s correlation coefficient for all prey identified by the 17 prey proteins. The analysis was done using ProHits-*viz.* Gene ontology enrichment analysis of the heatmap’s framed section. Data was obtained using ShinyGO (version 0.76) using a false discovery rate cut-off of 0.05 combined and the GO biological process pathway database. *B-E*, proximal associations for MCM2, POLE3, SSRP1, and RPA2. *Scatter plots* represent SAINT analyses per bait, using the untransfected 293FT negative control (n = 3 biological replicates). Prey proteins were considered to be proximal association partners if the SAINT score was ≥0.95 and the fold change over negative controls ≥1.5. The *colored dots* further denote prey proteins with a fold change enrichment ≥1.5 in over the GFP and GFP-NLS controls. Labeled proteins represent nuclear prey proteins identified using at least two negative controls. *Scatter plots* for the remaining prey proteins are found in supplemental Figs S3–S6. A complete list of the prey proteins and the confidence scores is available in the Supplemental Tables (Excel files). RPA, replication protein A; NLS, nuclear localization sequence.

A label-free quantitative MS approach was used to identify proximal associations for each protein from the different functional groups described above. Some of the negative controls resulted in overly stringent SAINT analyses. For example, only three MCM2 proximal associations were enriched over the GFP control; however, the same SAINT analysis using 293FT as the control identified >200 proximal associations, including five of the six subunits of the MCM2-7 hexameric ring and an expected proximity to factors such as ORC proteins, FACT, RPA, and DNA polymerases (Fig. 3 and supplemental Table S9). Keeping with this example, the topmost ontology (BP) term for all of the MCM2 proximal associations was “mitotic DNA replication” and related terms followed. For that reason, SAINT analyses were carried out using the 293FT control, but we nevertheless still compared fold enrichments over GFP and GFP-NLS. On the other hand, protein relative abundance fold change on treated condition was compared to the experimentally paired GFP-NLS-BioID2 control.

**Fig. 4:**
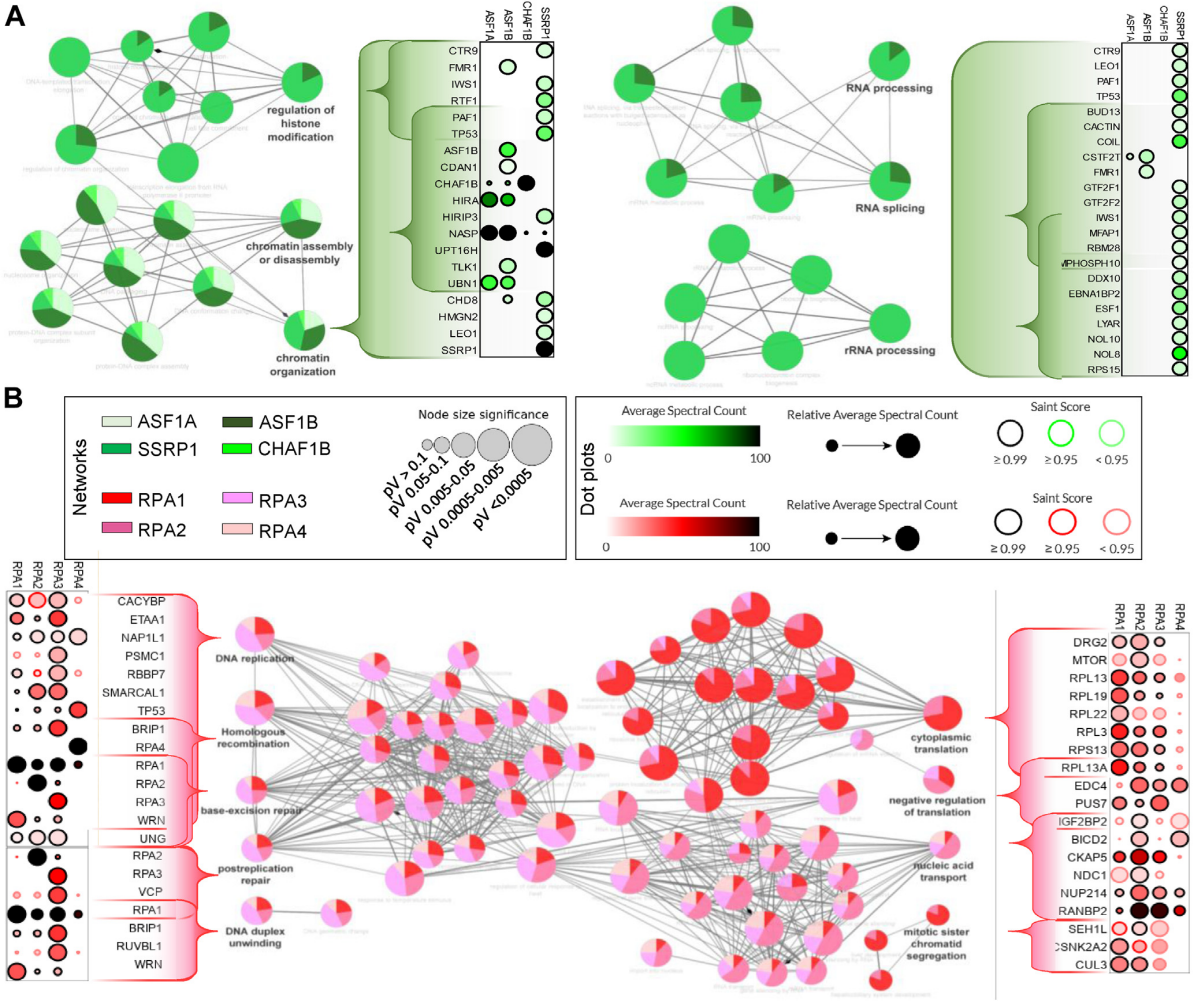
**Functional proteomic network for histone chaperones and RPA.** A functional enrichment analysis based on biological process (BP) and molecular function (MF) ontology terms was performed for the chromatin remodeler (*A*) and RPA bait categories within our analysis (*B*). The relative contribution of each bait toward shared terms is indicated in the *pie charts*. The radius of each *pie chart* is proportional to the significance of the terms. Proximal associations within key terms are shown as *dot plots* generated using ProHits-*viz.* (20). The *dot plots* show the relative abundance of prey nuclear proteins identified by BioID amongst the different baits. The color gradient is indicative of the average spectral counts per prey, and the edge color represents their SAINT score. RPA, replication protein A; BioID, biotin identification.

**Fig. 5:**
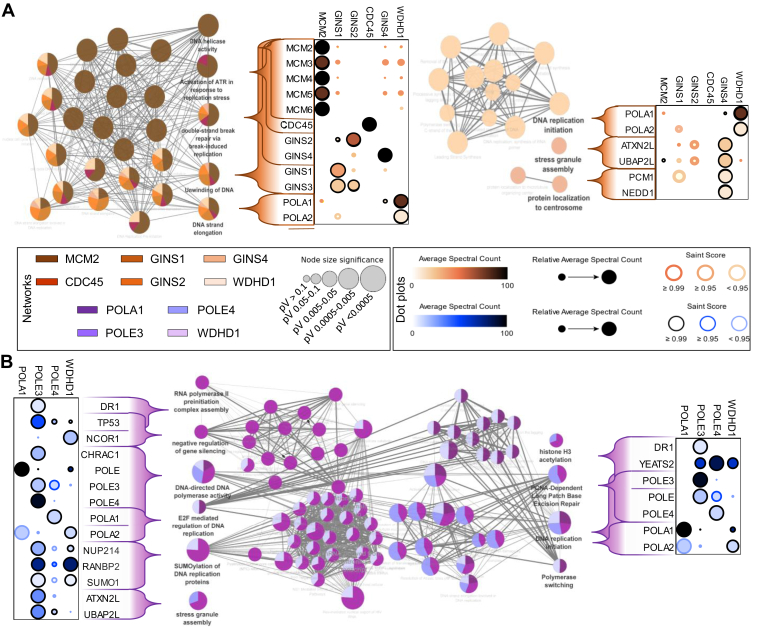
**Functional proteomic network for the CMG complex and DNA polymerases.** Functional enrichment analysis for the CMG complex (*A*) and DNA polymerases bait categories within our analysis (*B*). Data is presented as described in Figure 4. CMG, CDC45/MCM2-7/GINS.

The global interaction landscape of our 17 bait proteins was compared based on the prey MS/MS counts using Pearson’s correlation coefficients. The results, shown as a heat map (Fig. 3*A*), identified protein clusters containing proteins that likely colocalize or are part of the same complexes. The pathway enrichment analysis of the four more intense clusters was examined using Kyoto Encyclopedia of Genes and Genomes pathway database with ShinyGO v0.741 and revealed an enrichment of ribosome pathway (cluster A: FC = 49,6 and FDR = 1,6.10^−9^), nonhomologous end-joining pathway (cluster B: FC = 87,7 and FDR = 3,6.10^−2^), mismatch repair pathway (cluster C: FC = 48,3 and FDR = 5,2.10^−3^), and tight junction pathway (cluster D: FC = 10.8 and FDR = 3,9.10^−2^). Moreover, a more detailed enrichment function analysis is provided for cluster C, where DNA replication and DNA repair factors are represented, as expected. Indeed, pathways involved in mismatch repair, homologous recombination, DNA replication, and nucleotide excision repair were found enriched. Protein–protein associations for the CMG complex, polymerases, histone chaperones, and RPA is shown in Figure. 3, *B*–*E*, and supplemental Figs. S3–S6. Not all prey proteins could be labeled in the plots but the complete datasets are found in the Supplementary Tables. Key protein associations identified by us were further displayed as dot plots and interaction networks, highlighting functional enrichments (Figs. 4 and 5).

As mentioned above, the MCM2 bait identified several members of the MCM2-7 subcomplex (Fig. 5*A*), indicating that it was properly incorporated into the helicase complex. Likewise, the GINS subunits were strongly associated with one another. The proximity of some proteins could be deduced from the intensity of the BioID associations. For example, the crystal structure of the GINS subcomplex shows that GINS1 directly binds GINS3 and GINS4 (48, 49), and GINS3 is indeed one of the topmost prey proteins identified by GINS1, after GINS1 itself. GINS2 also directly binds GINS3 and, indeed, GINS3 was again the main hit after GINS2 (Figs. 5*A* and supplemental Fig. S4*D*). Likewise, GINS4 was associated with WDHD1 and WDHD1 was associated with POLA while the POLE BioID showed direct associations with the GINS complex, as previously reported (50, 51, 52) (Fig. 5*B* and supplemental Fig. S6*C*). The proximal associations with other (non-GINS) proteins are therefore highly intriguing and informative and open the door to further studies. For example, POLE3 and POLE4 are strongly associated with YEATS2, a scaffolding subunit of the ATAC complex that has acetyltransferase activity toward histones H3 and H4 and BAZ1A, a bromodomain-containing subunit of the ACF ATP-dependent chromatin remodeling complexes modulate nucleosome spacing (53, 54) (Fig. 3*C* and supplemental Fig. S6*B*). In the same vein, the tyrosine-protein kinase BAZ1B was also previously identified by Srivastava *et al.* (55) using BioID to probe the PCNA interactome with or without replication stress, where stress reorganized PCNA proximal associations such as those with BAZ1B. BAZ1A and YEATS2 are both involved in DNA replication and this highlights specialized roles conferred by various protein associations likely regulated in a spatiotemporal manner. The POLA1 bait failed to yield informative data, perhaps as a consequence of the protein fusion, but all other baits were highly informative and, as exemplified by the proteins above, by POLA identification as a prey when using other bait proteins (Fig. 5*B*).

The proteomic analysis of the histone chaperones recapitulated much of the established protein interactions but also revealed intriguing proximal associations. Most of the functional enrichment terms associated with these four proteins included regulation of histone modifications, chromatin assembly, chromatin organization, RNA processing, and RNA splicing, consistent with their functions. Interactions with several well-known histone deposition proteins were identified, such as NAP1, CAF-1 for replication-dependent histone deposition, and HIRA histone chaperone complex for replication-independent histone deposition (56, 57, 58, 59). Although ASF1A and ASF1B share some functional redundancy, they also exhibit tissue-specific and functional differences (60). There were interesting differences in the BioID datasets that likely reflect preferential associations with either ASF1A or ASF1B. It was intriguing, for example, to see a greater association between ASF1B and the TLK1 kinase (Fig. 4*A* and supplemental Table S2) that regulates ASF1 activity (61). CHD8 was also preferentially associated with ASF1B (Fig. 4*A*). Fewer proximal associations with CHAF1B/p60 and CDC45 were identified among all the proteins experimentally tested, although several known interactors were still found with lower SAINT score. This may have been the result of a less efficient labeling for these two proteins. FACT is also involved in gene transcription and DNA repair (44, 62), and the SSRP1 BioID prominently featured transcription elongation factors, such as the components of the PAF complex and chromatin remodeling proteins (Fig. 4*A*).

RPA is a heterotrimeric complex composed of RPA1-2-3, with important roles in several processes involving ssDNA (4, 63, 64, 65, 66). The RPA proteome was recently examined by us in the context of DNA repair but is here explored anew in a broader context (17). Like FACT, the RPA complex also associates with protein with a broad range of functions and those were very well captured by the BioID. RPA1 is notably known to interact with POLA1, per its role in replication, and that association was indeed recapitulated here (supplemental Table S5) (67). There were, however, several prey proteins that were commonly identified using the RPA1-2-3 bait proteins that are not only involved in DNA replication but also DNA repair and other aspects of nucleic acid regulation, and we observed significant differences with the interactome of RPA4, underlining a distinct role of the Alt–RPA complex (Fig. 4*B*). Although the canonical RPA complex has been extensively studied, the Alt–RPA complex where RPA4 replaces RPA2 is thus far only mentioned in a few studies (17, 35, 36, 68, 69, 70, 71). Our RPA4 BioID again offers the opportunity to better understand its functions. Consistent with the literature, RPA2 and RPA4 are mutually exclusive and we did not find them interacting with each other at all (Fig. 4*B*) (36). This is an important distinction that speaks to the need to better understand the RPA and Alt-RPA interactomes. One of the most striking differences was a pronounced RPA4 proximal association with TP53. Conversely, several preferential protein associations with canonical RPA were either greatly reduced or absent with RPA4, such as ETAA1, RBBP7, BRIP1, WRN, and UNG—which are involved in DNA replication and repair (Fig. 4*B*).

### Changes in the DNA Replication Proteome Following Treatment With HU

We next sought to identify changes in the replisome proximal associations upon HU-induced replication fork stalling. Here, only the GFP-NLS-BioID2 control was used to filter nonspecific nuclear protein associations for the strength and proximal association confidence it gives. As shown in supplemental Fig. S7*A*, the proximal associations with GFP-NLS-BioID2 had a near-perfect Pearson correlation coefficient (0.9876) when comparing untreated and HU-treated cells, meaning that HU had little impact on background associations. The untreated GFP-NLS-BioID2 control was therefore used to calculate the enrichment ratios for our prey proteins.

**Fig. 6:**
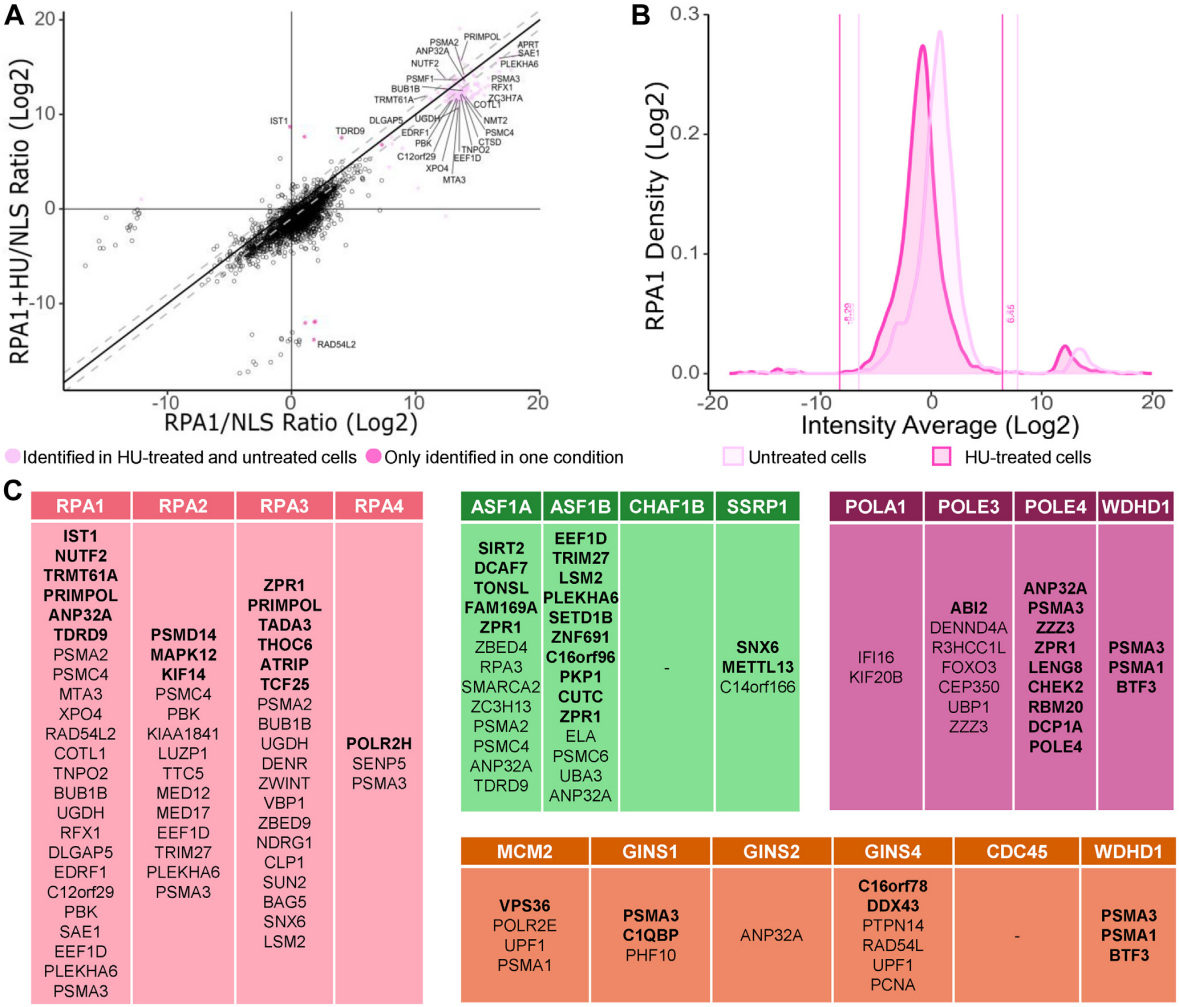
**Hydroxyurea-induced proteomic changes at replication forks.***A*, *scatter plot* comparing enrichment ratios for RPA1 prey proteins in untreated and HU-treated samples. *B*, distribution of enriched RPA1 prey proteins with a log2 fold change were identified using the two thresholds determined by the interquartile method. This method identifies proteins that were enriched in only one of the two analyzed conditions when greater to the positive or the negative threshold. The *graphic representations* for the other baits are shown in supplemental Figs. S8–S15. *C*, list of proximal nuclear protein associations affected by the hydroxyurea treatment. *Bold font* denotes gains while the remaining protein associations were depleted. RPA, replication protein A.

**Fig. 7:**
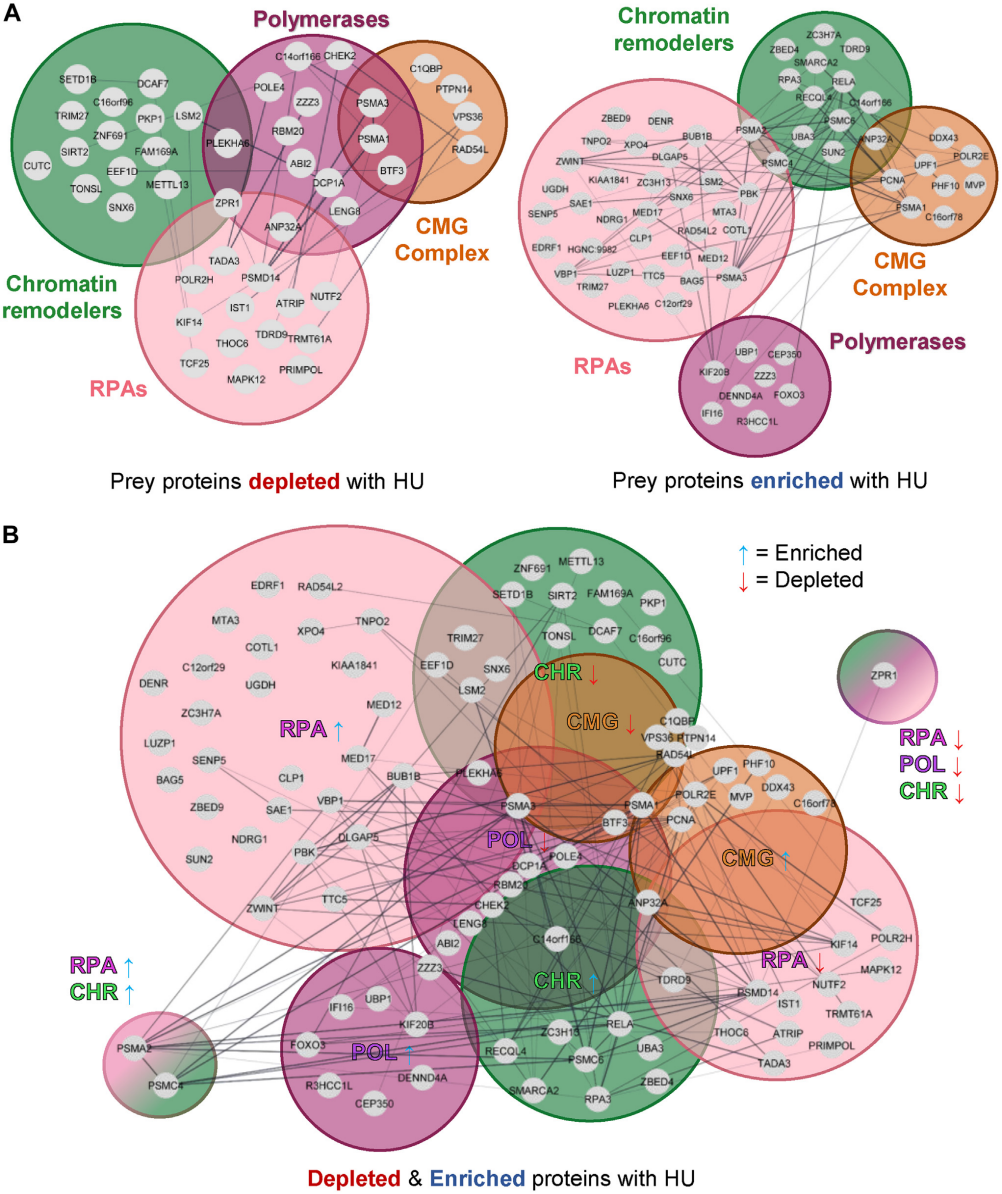
**Changes in the proximal association network during replicative stress.***A*, global replisome interaction network showing gained and depleted protein proximal associations in the presence of hydroxyurea. *B*, interaction network in the presence of hydroxyurea showing the interactome-dependent status of enriched and depleted common proteins. RPA, proteins identified from RPAs BioID bait complex; CHR, proteins identified from chromatin remodeler’s BioID baits complex; POL, proteins identified from polymerases BioID baits complex; CMG, proteins identified from CMG BioID baits complex. BioID, biotin identification; CMG, CDC45/MCM2-7/GINS; RPA, replication protein A.

To identify changes in the replisome proximal associations, we next plotted the enrichment ratios (replisome prey proteins over the GFP-NLS-BioID2 prey proteins) for untreated *versus* HU-treated cells (NT *versus* HU). An example is provided for RPA1 in Figure 6*A* and the analysis for RPA, CMG, chromatin remodelers, and polymerase proteins are respectively shown in supplemental Figs. S8, S10, S12, and S14. Supplemental Fig. S7*B* summarizes the number of unique peptides and percentage sequence coverage for our bait proteins throughout the various MS analyses. To avoid identifying unspecific proteins association regardless of the treatment, the proteins had to be identified as interactors either with or without treatment with HU (Fig. 6*B* for RPA1 and supplemental Figs. S9, S11, S13, and S15 for RPA, CMG complex, chromatin remodelers, and polymerases proximity association distributions, respectively). The analysis identified proteins that were either recruited (bold font) or depleted (normal font) from the replisome when cells were treated with HU (Fig. 6*C*). To simplify the list, we only included nuclear proteins and proteins of unknown/poorly annotated functions. Venn diagrams further highlight the altered associations across each functional cluster (Fig. 7).

RPA proteins had the greatest proteomic changes when cells were treated with HU, which is consistent with an accumulation of ssDNA. Persistently stalled and unresolved DNA replication forks generate double-stranded breaks and can induce signaling through both ataxia telangiectasia and Rad3-related (ATR) and ataxia telangiectasia mutated. The canonical pathway triggers the binding of the RPA complex to ssDNA, which then serves as a platform for the recruitment of many other proteins including the ATR-interacting protein. That in turn facilitates the recruitment of yet other DNA repair factors (72, 73, 74). Our experiments confirm established RPA interactors such as fork processing ssDNA binding proteins (*e.g.*, BLM, WRN, BRCA2, and RAD51) but also revealed novel interactions modulated by HU (*e.g.*, TDRD9, C12orf29, and ZPR1). Interestingly, Alt-RPA (RPA4) showed much less difference in its interactome upon replication fork stalling, suggesting functional divergence from the canonical RPA1 to 3 complex.

**Fig. 8:**
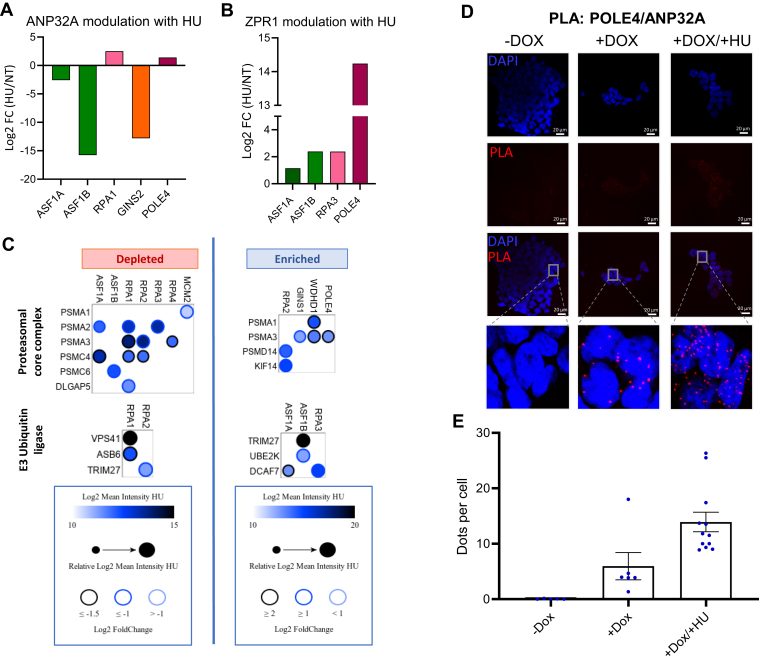
**Quantitative analysis of altered protein associations upon DNA damage.***A* and *B*, histograms comparing the fold change (FC) enrichment of ANP32A and ZPR1 spectra when treating cells with HU. *C*, *dot plots* showing changes in proteasome and E3 ubiquitin ligase protein associations with replisome components in the presence of hydroxyurea. Data is plotted as explained in Figure 4. *D*, visualization of proximity association with endogenous ANP43A and the POLE4 bait (FLAG) by proximity ligation assay (PLA). POLE4 293FT cells were induced for 48 h with doxycycline and either left untreated or exposed to 500 μM of HU for 24 h. Nuclei were counterstained with 4′,6-diamidino-2-phenylindole. *E*, quantification of PLA signals using ImageJ. Data is shown as the mean ± SEM from at least 92 cells.

Amongst all the proteins identified, the Acidic leucine-rich nuclear phosphoprotein 32 family member A (ANP32A) was commonly identified by various baits (Fig. 8*A*). Interestingly, its associations were greatly modulated by HU. The latter causes its depletion from the polymerase and increased associations with RPA and polymerases, exemplifying some of the reorganizations that occur within replisomes upon stress. Some of these changes were validated by PLA (Figs. 8, *A*, *B*, *C*, *D*, and *E*, and supplemental Figs. S16). ANP32A is a multifunctional nuclear protein with roles in transcriptional regulation, modulation of histone acetylation, chromatin remodeling, mRNA export, and cell death (75). Within the INHAT complex, ANP32A notably regulates acetylation levels on newly synthesized histone H4 by opposing acetylation by HAT1 (76). The proteomic data hence suggests that HU impacts the predeposition histone marks through various means, which is in line with prior reports (77, 78).

Other notable changes were observed upon HU treatment. There was, for example, an increased association between ANP32A with POLE4 in response to replicative stress showing a reorganization of association strengths at forks (Fig. 8, *A*–*D*). Changes in associations were also observed. Several proteasomal subunits were notably found to interact with components of the CMG and polymerase complexes, suggesting yet another level of protein regulation at stalled replication forks (Fig. 8*C*). Indeed, in the absence of fork progression, the CMG complex is ubiquitylated and leads to its degradation (79). A slight loss of CMG proteins and other replication components was observed in our immunoblots for HU-treated samples (supplemental Fig. S1, *E*–*U*). ZPR1 is another interesting example of fork reorganization identified by our proteomic screen. ZPR1 is a cytoplasmic zinc finger protein that translocates to the nucleus in S phase (80). It is a modifier of spinal muscular atrophy recently found to counter an accumulation of R loops (81). Our data shows ZPR1 proximal associations with ASF1A, ASF1B, RPA3, and POLE4 that are greatly enhanced upon HU-induced fork stalling (Fig. 8*B*). The increased ZPR1 associations with ASF1B and POLE4 were also observed by PLA (supplemental Fig. S16, *B* and *D*). Even if a role as a protective modifier of spinal muscular atrophy is established for ZPR1, the results open the door to further molecular characterization of this protective mechanism. Altogether, our proteomic dataset provides a rich resource amenable to various new investigation venues on DNA replication and repair.

## Discussion

We defined the interactome of 17 replisome proteins that fall within four functional categories and the impact of fork stalling. By following multiple subunits of the same components by BioID, we were able to increase the confidence within our proteomic screen, gain insights into the organization of replication forks, and assess changes caused by HU. Our experimental pipeline was designed so to concomitantly expose cells to HU and biotin and ensure that the prey proteins fully captured the dynamic changes caused by HU rather than its endpoint. The complete BioID dataset showed the expected functional enrichment in DNA replication, cell cycle control, and DNA damage response proteins (Figs. 4 and 5). A comparison of this BioID dataset with other large-scale studies such as BioPlex (82), BioGRID (83), and OpenCell (84) showed up to a 25% overlap for the combined 17 bait proteins, despite the differences in the approaches and in the cut-offs used to define interactors. This overlap reached up to 80% for some bait proteins such as ASF1A, where nearly all of our BioID hits were also identified by the other databases, confirming established associations. The identification of additional proximal associations hence complete existing databases and provides further information on fork dynamics, notably upon HU exposure.

Several innovative methods have been developed in the past decade to study proteins involved in DNA replication such as isolation of proteins on nascent DNA (iPOND) and nascent chromatin capture (NCC). Coupled with MS, these have probed the proteomic composition of genomic sites containing nascent DNA under both normal and stress conditions (85, 86, 87, 88). Both techniques use thymidine analogs to label and isolate newly replicated DNA and the associated proteins. Our approach is complementary but different, in which we probed proximal association with proteins involved in DNA unwinding, ssDNA binding, DNA replication, and histone deposition. The overlaps and differences among the different approaches are informative and emphasize the benefit of using combinatorial approaches when studying complex processes such as DNA replication and repair.

As mentioned above, BioID is advantageous in that it identifies protein associations regardless of whether they are direct or not and regardless of whether they are biochemically stable or labile (89). Nevertheless, the intersections between our BioID and iPOND (87) or NCC enrich for nuclear proteins with annotations in DNA replication fork and chromatin organization, as well as DNA repair in the presence of HU (supplemental Fig. S17, *A* and *B*). These shared proteins identified represent a total of 21.3% of the 347 nuclear proteins enriched in our study, a sizeable coverage considering that these approaches are very different from a technical point of view and probe different components (replication proteins *versus* proteins on newly replicated DNA). Past iPOND and NCC studies have also measured the changes in protein proximity association, following DNA replication stress using HU (86, 87, 90). The overlap between our BioID and these was limited to nine proteins (supplemental Fig. S17*B*). We attribute the limited overlap to technical differences since iPOND and NCC captured interactions on nascent DNA after a brief exposure to a high dose of HU (IPOND: 3 and 5 mM HU from 5 min to 24 h/NCC: 3 mM for 30 min), while here, the 17 BioID interactomes capture protein-dependent interactions over a 24-h 1 mM HU treatment.

Replication fork dynamics should be taken into consideration since some bait proteins were not expected to solely be present at sites of active DNA replication. Only a few MCM2-7 rings at dormant origins of replication would fire and assemble into an active CMG helicase, for example (91, 92). Likewise, FACT also enriches at transcription sites (93, 94). Moreover, there are intrinsic limitations to all techniques and it should be noted that BioID cannot distinguish between posttranslational changes in protein associations from those due to altered protein expression (*i.e.*, when comparing untreated and HU-treated cells). This is why we verified and confirmed that HU had little influence on the control BioIDs (supplemental Fig. S7*A*). Finally, even though our datasets enriched for the expected proteins and ontology terms, some replication factors did not make our statistical cut in this analysis. There was a notable lack of PCNA in the CAF-1 p60 BioID even though CAF-1 p150 was identified, suggesting that the biotin ligase was perhaps not optimally positioned to capture a broader range of associations. Despite these limitations, the BioID dataset remains quite powerful and identified important features not captured by traditional affinity purifications worthy of further investigation.

Stalled replication forks are either stabilized and rescued (*e.g.*, *via* a converging fork or the recruitment of enzymes that facilitate fork restart) or disassembled to prevent replication fork collapse (95, 96). Whether the stalled forks recover or collapse depends on tightly regulated processes. These entail their protection from nucleolytic degradation, removal of the blocking lesions or their bypass *via* translesion synthesis or repriming, and ultimately RAD51-mediated replication restart (97, 98). Ubiquitylation plays a key role in regulating these fork-associated processes, including K63-linked polyubiquitin chains and PCNA polyubiquitylation (98, 99). We identified several subunits of the 26S proteasome as BioID prey proteins and observed a reorganization of these associations upon HU treatment (Fig. 8*C*). This is interesting because it indicates that replication fork stalling induced by HU causes perhaps not only the disassembly of these complexes at the site of replication but can also target them for degradation. Many ubiquitin signaling network proteins were identified by our baits, including DCAF7, TRIM27, UBE2K, VPS41, and ASB6. DCAF7, a component of an E3 ligase, targets DNA ligase I (100) and its identification in the HU BioID hints at a regulatory role in the replication stress response, as an example.

The heterotrimeric RPA complex is an important player in almost all DNA metabolic pathways (namely replication, repair, recombination, and damage checkpoints) and is highly enriched at stalled replication forks that collapse (66). In line with such a role, we found HU to cause an enrichment of RPA with DNA repair factors such as BLM, WRN, BRCA2, ATR-interacting protein, XPG, RECQL, and SMARCAL1, as well as many other RPA-interacting proteins. This is also consistent with an extensive production of nascent-strand ssDNA at replication forks in HU-treated cells, and the need for fork reversal, enzymatic cleavage, and end resection (90, 101, 102).

A particularly interesting aspect of our study was the identification of proteins that were neither known to play a role in DNA replication nor repair, again hinting at uncharacterized functions, and opening the door to further studies. Interestingly, many of these poorly characterized proteins shared important structural features such as nucleic acid–binding zinc fingers (*e.g.*, ZZZ3 and ZPR1) and methyl-binding Tudor domains (*e.g.*, the TDRD9 RNA helicase, and the multifunctional ANP32A protein discussed above). Many of the newly identified prey proteins remain nearly completely uncharacterized (*e.g.*, C12orf29/RLIG1 recently found to repair RNA (103), C16orf78, and C16orf96 a putative risk gene for sarcomas (104)), warranting further investigations. Some of these HU-enhanced associations were verified (supplemental Fig. S16). For example, HU caused a switch in ANP32A associations, depleting those with histone chaperones and the GINS complex and enriching the ones with RPA and DNA polymerases. ANP32A is required for influenza viral genome replication (105), but its role in mammalian DNA replication needs further elucidation. Indeed, ANP32A was also identified in nascent DNA proteome databases (85, 88, 106, 107), and future work is needed to address its roles and the intriguing nature of the HU-induced association switch.

Collectively, our multisubunit BioID study of the replisome provides a powerful discovery approach with a broad multidimensional coverage. We have delineated how the different subunits interplay during DNA replication, and we have investigated the molecular consequences associated with the stalling of the replication fork. This revealed novel interconnections among various factors with key functions in DNA replication.

## Data Availability

The MS proteomics data have been deposited to the ProteomeXchange Consortium *via* the PRIDE (108) partner repository with the dataset identifier PXD037136.

## Supplemental Data

This article contains supplemental data (86, 87).

## Conflict of interest

The authors declare no competing interests.

## Supplemental Data

Supplemental Table S1 Supplemental Tables S1–S17 (Excel files): Complete BioID dataset from untreated and hydroxyurea-treated cells. Each file contains the SAINT and Prostar data for one of the 17 bait proteins. The first three Excel tabs contain SAINT data respectively using the 293FT, GFP, and GFP-NLS controls. The fourth tab shows the interactome data from untreated cells. The fifth tab shows Prostar data after the removal of missing values (as described in the statistical analysis section), while the sixth tab contains the list of proteins whose association with the replisome was either depleted or enriched in HU-treated cells.

Supplemental Table S17

Supplemental Figure S1 Hydroxyurea-induced DNA damage in 293FT cells and bait protein expression. *A*, 293FT cells were treated with increasing hydroxyurea concentrations for 24 h. The extent of DNA damage was analyzed by immunoblotting using a γ-H2AX antibody. *B*, Immunolabeling of HU-treated HEK293 Flp-In T-REx (293FT) cells showing γ-H2AX accumulation 24 hrs after treatment. Scale bars = 10 μm. *C*, Flow cytometry analysis of propidium iodine-stained cells with or without exposure to 1 mM hydroxyurea for 24 h (n = 3). *D*, Quantification of the cell proportion in each phase of the cell cycle. The experiments were independently repeated three times, and the data was presented by mean ± standard deviation (SD). Statistical analyses were done using a two-way ANOVA test, with *p* < 0.05 considered as significantly different (ns = non-significant, ∗∗*p* < 0.001). *E*–*U*, Expression of the different BioID2 fusion proteins verified by immunoblotting. Isogenic 293FT cells expressing each of the bait proteins were induced with doxycycline for 48 h and detected using Myc or Flag epitope tags. The γH2AX marker was used as a surrogate for DNA damage in cells exposed to 1 mM Hydroxyurea for 24 h. The expressed bait protein is indicated over each set of blots. **Supplemental Figure S1**: Immunofluorescent labeling of bait proteins showing their sub-cellular localization. Isogenic HEK293 Flp-In T-REx cells were induced with doxycycline for 48 h and bait proteins detected using antibodies against either Myc or Flag tags. The expressed bait protein is indicated over each set of micrographs. Scale bars = 10 μm. **Supplemental Figure S3**: Scatter plots showing the proximal associations for RPA proteins in untreated cells. Prey proteins were considered enriched over the 293FT control if the SAINT score ≥0.95. The colored dots further denote prey proteins with a fold change enrichment ≥1.5 in over the GFP and GFP-NLS controls. Labeled proteins represent nuclear prey proteins identified using at least two negative controls. Data represents three biological replicates for all samples. A complete list of the prey proteins and the confidence scores is available in the supplemental Tables S5, S7, and S8. **Supplemental Figure S4**: Scatter plots showing the proximal associations for CMG subunits in untreated cells. Data is presented as explained in supplemental Fig. S4. A complete list of the prey proteins and the confidence scores is available in the supplemental Tables S9, S10, S11, S12, S13, and S17. **Supplemental Figure S5**: Scatter plots showing the proximal associations for the histone chaperones in untreated cells. Data is presented as explained in supplemental Fig. S4. A complete list of the prey proteins and the confidence scores is available in the supplemental Tables S1–S3. **Supplemental Figure S6**: Scatter plots showing the proximal associations for the polymerases in untreated cells. Data is presented as explained in supplemental Fig. S4. A complete list of the prey proteins and the confidence scores is available in the supplemental Tables S14, S16, and S17. **Supplemental Figure S7**: Prey proteins reciprocally identified across our 17 replisome bait proteins. *A*, Comparison of the BioID data obtained using the BioID2-GFP-NLS control in HU-treated and untreated cells. Minimal changes were observed in these background proximal associations, per the high Pearson correlation coefficient (r). Untreated GFP-NLS BioID2 controls were thus used to analyze BioID data from HU-treated cells. *B*, Unique peptides and sequence coverage (%) for bait proteins that were also identified as prey across the various BioID experiments. **Supplemental Figure S8**: HU-induced changes in RPA proximal associations. Protein intensity levels were normalized using the mean intensity levels from the GFP-NLS control in untreated cells. Light-colored points represent enriched proteins whose log2 foldchange lies within the two thresholds determined by the interquartile method, meaning that they were common to both conditions. Dark-colored points represent proteins whose log2 foldchange values were lower or higher than the determined threshold (see supplemental Fig. S10), meaning that they were enriched in one condition only. Labeled proteins are high-confidence nuclear prey proteins used in downstream analyses of the replisome in response to hydroxyurea. **Supplemental Figure S9**: RPA BioID2 distributions and threshold assignment using an interquartile method. Protein intensity levels were normalized using the mean intensity levels from the GFP-NLS control in untreated cells. Proteins with intensity levels greater than 1 in both conditions (HU/NLS and NLS) and less than or greater than Q1 - 1,5 × (Q3 - Q1) and Q3 + 1,5 × (Q3 - Q1) values were considered to be modulated by HU (interquartile range methods). **Supplemental Figure S10**: CMG complex proteome changes caused by hydroxyurea. Data was normalized and plotted as explained in supplemental Fig. S8. Thresholds are defined in supplemental Fig. S11. **Supplemental Figure S11**: CMG BioID2 distributions and threshold assignment using an interquartile method. Data was plotted in supplemental Fig. S10 in the same manner as explained in supplemental Fig. S9. **Supplemental Figure S12**: Histone chaperone proteome changes caused by hydroxyurea. Data was normalized and plotted as explained in supplemental Fig. S8. Thresholds are defined in supplemental Fig. S13). **Supplemental Figure S13**: Histone chaperone BioID2 distributions and threshold assignment using an interquartile method. Data was plotted as explained in supplemental Fig. S12 in the same manner as explained in supplemental Fig. S9. **Supplemental Figure S14**: Polymerase complex proteome changes caused by hydroxyurea. Data was normalized and plotted as explained in supplemental Fig. S8. Thresholds are defined in supplemental Fig. S15. **Supplemental Figure S15**: Polymerase BioID2 distributions and threshold assignment using the interquartile method. Data was plotted as explained in supplemental Fig. S14 in the same manner as explained in supplemental Fig. S9. **Supplemental Figure S16**: Validation of the ANP32A and ZPR1 associations with replisome proteins. Interactions between bait proteins and endogenous ANP32A and ZPR1 were visualized by PLA. Flag-BIOID2-GINS2, Flag-BIOID2-POLE4, and 3xMyc-BIOID2-ASF1B expression were induced with doxycycline for 48 h. Cells were treated with 500 μM of HU for 24 h to block replication. *A*, Micrographs showing Flag-BIOID2-GINS2 + ANP32A proximal associations. PLA signal numbers were quantified using ImageJ and plotted as the mean ± SEM from a minimum 69 cells. *B*, PLA data for Flag-BioID2-POLE4 + ZPR1 collected from 216 cells. *C*, PLA data for 3xMyc-BioID2-ASF1B + ANP32A collected from 10 cells. *D*, PLA data for 3xMyc-BioID2-ASF1B + ZPR1 PLA data collected from 7 cells. **Supplemental Figure S17**: Venn diagram comparing the BioID for untreated/HU-treated cells with iPOND and NCC previously published results. Comparison of BioID2-MS dataset with two other comprehensive proteomic analyses of HU arrested or damaged replication forks by HU (iPOND-SILAC-MS dataset from Dundrawala *et al.*, 2015 and NCC-SILAC-MS from Nakamura *et al.*, 2021). Results are depicted as Venn diagrams comparing identified proteins in *A*, normal conditions, and (*B*) HU-treated cells. Significance thresholds were uniquely defined by each study and left as such. A gene ontology enrichment analysis was done for the intersections that overlapped with our BioID2-MS analysis.

## References

[1] Costa A., Ilves I., Tamberg N., Petojevic T., Nogales E., Botchan R.M. (2011). The structural basis for MCM2–7 helicase activation by GINS and Cdc45. Nat. Struct. Mol. Biol..

[2] Jones M.L., Baris Y., Taylor M.R.G., Yeeles J.T.P. (2021). Structure of a human replisome shows the organisation and interactions of a DNA replication machine. EMBO J..

[3] Costa A., Diffley J.F.X. (2022). The initiation of eukaryotic DNA replication. Annu. Rev. Biochem..

[4] Wold M.S. (1997). Replication protein A: a heterotrimeric, single-stranded DNA-binding protein required for eukaryotic DNA metabolism. Annu. Rev. Biochem..

[5] Willhoft O., Costa A. (2021). A structural framework for DNA replication and transcription through chromatin. Curr. Opin. Struct. Biol..

[6] Foltman M., Evrin C., De Piccoli G., Jones R.C., Edmondson R.D., Katou Y. (2013). Eukaryotic replisome components cooperate to process histones during chromosome replication. Cell Rep..

[7] Gambus A., Van Deursen F., Polychronopoulos D., Foltman M., Jones R.C., Edmondson R.D. (2009). A key role for Ctf4 in coupling the MCM2-7 helicase to DNA polymerase α within the eukaryotic replisome. EMBO J..

[8] Gan H., Serra-Cardona A., Hua X., Zhou H., Labib K., Yu C. (2018). The Mcm2-Ctf4-Polα Axis facilitates parental histone H3-H4 transfer to lagging strands. Mol. Cell.

[9] Bellelli R., Belan O., Pye V.E., Clement C., Maslen S.L., Skehel J.M. (2018). POLE3-POLE4 is a histone H3-H4 chaperone that maintains chromatin integrity during DNA replication. Mol. Cell.

[10] Smith S., Stillman B. (1989). Purification and characterization of CAF-I, a human cell factor required for chromatin assembly during DNA replication *in vitro*. Cell.

[11] Tyler J.K., Collins K.A., Prasad-Sinha J., Amiott E., Bulger M., Harte P.J. (2001). Interaction between the Drosophila CAF-1 and ASF1 chromatin assembly factors. Mol. Cell. Biol..

[12] Groth A., Corpet A., Cook A.J.L., Roche D., Bartek J., Lukas J. (2007). Regulation of replication fork progression through histone supply and demand. Science.

[13] Hart T., Chandrashekhar M., Aregger M., Steinhart Z., Brown K.R., MacLeod G. (2015). High-resolution CRISPR screens reveal fitness genes and genotype-specific cancer liabilities. Cell.

[14] Bianchi V., Pontis E., Reichard P. (1986). Changes of deoxyribonucleoside triphosphate pools induced by hydroxyurea and their relation to DNA synthesis. J. Biol. Chem..

[15] Taylor M.R.G., Yeeles J.T.P. (2018). The initial response of a eukaryotic replisome to DNA damage. Mol. Cell.

[16] Kim D.I., Jensen S.C., Noble K.A., Kc B., Roux K.H., Motamedchaboki K. (2016). An improved smaller biotin ligase for BioID proximity labeling. Mol. Biol. Cell.

[17] Gall-Duncan T., Luo J., Jurkovic C.M., Fischer L.A., Fujita K., Deshmukh A.L. (2023). Antagonistic roles of canonical and alternative-RPA in disease-associated tandem CAG repeat instability. Cell.

[18] Wieczorek S., Combes F., Lazar C., Gianetto Q.G., Gatto L., Dorffer A. (2017). DAPAR & ProStaR: software to perform statistical analyses in quantitative discovery proteomics. Bioinformatics.

[19] Teo G., Liu G., Zhang J., Nesvizhskii A.I., Gingras A.C., Choi H. (2014). SAINTexpress: improvements and additional features in significance analysis of Interactome software. J. Proteomics.

[20] Knight J.D., Choi H., Gupta G.D., Pelletier L., Raught B., Nesvizhskii A.I. (2016). ProHits-viz: a suite of web-tools for visualizing interaction proteomics data. Nat. Methods.

[21] Shannon P., Markiel A., Owen O., Nitin S B., Jonathan T.,W., Daniel R. (2003). Cytoscape: a software environment for integrated models of biomolecular interaction networks. Genome Res..

[22] Bindea G., Mlecnik B., Hackl H., Charoentong P., Tosolini M., Kirilovsky A. (2009). ClueGO: a Cytoscape plug-in to decipher functionally grouped gene ontology and pathway annotation networks. Bioinformatics.

[23] Hulsen T., de Vlieg J., Alkema W. (2008). BioVenn - a web application for the comparison and visualization of biological lists using area-proportional Venn diagrams. BMC Genomics.

[24] Kang Y.H., Galal W.C., Farina A., Tappin I., Hurwitz J. (2012). Properties of the human Cdc45/Mcm2-7/GINS helicase complex and its action with DNA polymerase ε in rolling circle DNA synthesis. Proc. Natl. Acad. Sci. U. S. A..

[25] Kang S., Kang M.S., Ryu E., Myung K. (2018). Eukaryotic DNA replication: orchestrated action of multi-subunit protein complexes. Mutat. Res..

[26] Huang H., Strømme C.B., Saredi G., Hödl M., Strandsby A., González-Aguilera C. (2015). A unique binding mode enables MCM2 to chaperone histones H3-H4 at replication forks. Nat. Struct. Mol. Biol..

[27] Mcquaid M.E., Ahmed K., Tran S., Rousseau J., Shaheen R., Kernohan K.D. (2022). Hypomorphic GINS3 variants alter DNA replication and cause Meier-Gorlin syndrome. JCI Insight.

[28] Moyer S.E., Lewis P.W., Botchan M.R. (2006). Isolation of the Cdc45/Mcm2-7/GINS (CMG) complex, a candidate for the eukaryotic DNA replication fork helicase. Proc. Natl. Acad. Sci. U. S. A..

[29] Zhu W., Ukomadu C., Jha S., Senga T., Dhar S.K., Wohlschlegel J.A. (2007). Mcm10 and And-1/CTF4 recruit DNA polymerase α to chromatin for initiation of DNA replication. Genes Dev..

[30] Bermudez V.P., Farina A., Tappin I., Hurwitz J. (2010). Influence of the human cohesion establishment factor Ctf4/AND-1 on DNA replication. J. Biol. Chem..

[31] Kilkenny M.L., Simon A.C., Mainwaring J., Wirthensohn D., Holzer S., Pellegrini L. (2017). The human CTF4-orthologue AND-1 interacts with DNA polymerase a/primase *via* its unique C-Terminal HMG box. Open Biol..

[32] Simon A.C., Zhou J.C., Perera R.L., Deursen F. V, Evrin C., Labib K. (2014). A Ctf4 trimer couples the CMG helicase to DNA polymerase α in the eukaryotic replisome. Nature.

[33] Aria V., Yeeles J.T.P. (2019). Mechanism of bidirectional leading-strand synthesis establishment at eukaryotic DNA replication origins. Mol. Cell.

[34] Lõoke M., Maloney M.F., Bell S.P. (2017). Mcm10 regulates DNA replication elongation by stimulating the CMG replicative helicase. Genes Dev..

[35] Kemp M.G., Mason A.C., Carreira A., Reardon J.T., Haring S.J., Borgstahl G.E.O. (2010). An alternative form of replication protein A expressed in normal human tissues supports DNA repair. J. Biol. Chem..

[36] Keshav K.F., Chen C., Dutta A. (1995). Rpa4, a homolog of the 34-kilodalton subunit of the replication protein A complex. Mol. Cell. Biol..

[37] Richet N., Liu D., Legrand P., Velours C., Corpet A., Gaubert A. (2015). Structural insight into how the human helicase subunit MCM2 may act as a histone chaperone together with ASF1 at the replication fork..pdf. Nucleic Acids Res..

[38] Liu W.H., Roemer S.C., Port A.M., Churchill M.E.A. (2012). CAF-1-induced oligomerization of histones H3/H4 and mutually exclusive interactions with Asf1 guide H3/H4 transitions among histone chaperones and DNA. Nucleic Acids Res..

[39] Liu W.H., Roemer S.C., Zhou Y., Shen Z.J., Dennehey B.K., Balsbaugh J.L. (2016). The Cac1 subunit of histone chaperone CAF-1 organizes CAF-1-H3/H4 architecture and tetramerizes histones. Elife.

[40] Mello J.A., Silljé H.H.W., Roche D.M.J., Kirschner D.B., Nigg E.A., Almouzni G. (2002). Human Asf1 and CAF-1 interact and synergize in a repair-coupled nucleosome assembly pathway. EMBO Rep..

[41] Zhang W., Zeng F., Liu Y., Shao C., Li S., Lv H. (2015). Crystal structure of human SSRP1 middle domain reveals a role in DNA binding. Sci. Rep..

[42] Gao Y., Li C., Wei L., Teng Y., Nakajima S., Chen X. (2017). SSRP1 cooperates with PARP and XRCC1 to facilitate single strand DNA break repair by chromatin priming.pdf. Cancer Res..

[43] Abe T., Sugimura K., Hosono Y., Takami Y., Akita M., Yoshimura A. (2011). The histone chaperone facilitates chromatin transcription (FACT) protein maintains normal replication fork rates. J. Biol. Chem..

[44] Liu Y., Zhou K., Zhang N., Wei H., Tan Y.Z., Zhang Z. (2020). FACT caught in the act of manipulating the nucleosome. Nature.

[45] Jensen L.J., Kuhn M., Stark M., Chaffron S., Creevey C., Muller J. (2009). String 8 - a global view on proteins and their functional interactions in 630 organisms. Nucleic Acids Res..

[46] Dickey J.S., Redon C.E., Nakamura A.J., Baird B.J., Sedelnikova O.A., Bonner W.M. (2009). H2AX: functional roles and potential applications. Chromosoma.

[47] Thul P.J., Akesson L., Wiking M., Mahdessian D., Geladaki A., Ait Blal H. (2017). A subcellular map of the human proteome. Science.

[48] Chang Y.P., Wang G., Bermudez V., Hurwitz J., Chen X.S. (2007). Crystal structure of the GINS complex and functional insights into its role in DNA replication. Proc. Natl. Acad. Sci. U. S. A..

[49] Choi J.M., Lim H.S., Kim J.J., Kyu S.O., Yunje C. (2007). Crystal structure of the human GINS complex. Genes Dev..

[50] Kamada K., Kubota Y., Arata T., Shindo Y., Hanaoka F. (2007). Structure of the human GINS complex and its assembly and functional interface in replication initiation. Nat. Struct. Mol. Biol..

[51] Galal W.C., Kang Y.H., Hurwitz J. (2012). Establishing the human rolling circle reaction. Cell Cycle.

[52] Langston L.D., Zhang D., Yurieva O., Georgescu R.E., Finkelstein J., Yao N.Y. (2014). CMG helicase and DNA polymerase ε form a functional 15-subunit holoenzyme for eukaryotic leading-strand DNA replication. Proc. Natl. Acad. Sci. U. S. A..

[53] Guelman S., Kozuka K., Mao Y., Pham V., Solloway M.J., Wang J. (2009). The double-histone-acetyltransferase complex ATAC is essential for mammalian development. Mol. Cell. Biol..

[54] Yang J.G., Madrid T.S., Sevastopoulos E., Narlikar G.J. (2006). The chromatin-remodeling enzyme ACF is an ATP-dependent DNA length sensor that regulates nucleosome spacing. Nat. Struct. Mol. Biol..

[55] Srivastava M., Chen Z., Zhang H., Tang M., Wang C., Jung S.Y. (2018). Replisome dynamics and their functional relevance upon DNA damage through the PCNA interactome. Cell Rep..

[56] Pchelintsev N.A., McBryan T., Rai T.S., VanTuyn J., Ray-Gallet D., Almouzni G. (2013). Placing the HIRA histone chaperone complex in the chromatin landscape. Cell Rep..

[57] Moshkin Y.M., Kan T.W., Goodfellow H., Bezstarosti K., Maeda R.K., Pilyugin M. (2009). Histone chaperones ASF1 and NAP1 differentially modulate removal of active histone marks by LID-RPD3 complexes during NOTCH silencing. Mol. Cell.

[58] Tang Y., Poustovoitov M.V., Zhao K., Garfinkel M., Canutescu A., Dunbrack R. (2006). Structure of a human ASF1a-HIRA complex and insights into specificity of histone chaperone complex assembly. Nat. Struct. Mol. Biol..

[59] Galvani A., Courbeyrette R., Agez M., Ochsenbein F., Mann C., Thuret J.-Y. (2008). *In Vivo* study of the nucleosome assembly functions of ASF1 histone chaperones in human cells. Mol. Cell. Biol..

[60] Abascal F., Corpet A., Gurard-Levin Z.A., Juan D., Ochsenbein F., Rico D. (2013). Subfunctionalization *via* adaptive evolution influenced by genomic context: the case of histone chaperones ASF1a and ASF1b. Mol. Biol. Evol..

[61] Pilyugin M., Demmers J., Peter Verrijzer C., Karch F., Moshkin Y.M. (2009). Phosphorylation-mediated control of histone chaperone ASF1 levels by tousled-like kinases. PLoS One.

[62] Orphanides G., LeRoy G., Chang C.H., Luse D.S., Reinberg D. (1998). FACT, a factor that facilitates transcript elongation through nucleosomes. Cell.

[63] Gomes X.V., Henricksen L.A., Wold M.S. (1996). Proteolytic mapping of human replication protein A: evidence for multiple structural domains and a conformational change upon interaction with single-stranded DNA. Biochemistry.

[64] Zou Y., Liu Y., Wu X., Shell S.M. (2006). Functions of human replication protein A (RPA): from DNA replication to DNA damage and stress responses. J. Cell. Physiol..

[65] Fan J., Pavletich N.P. (2012). Structure and conformational change of a replication protein A heterotrimer bound to ssDNA. Genes Dev..

[66] Caldwell C.C., Spies M. (2020). Dynamic elements of replication protein A at the crossroads of DNA replication, recombination, and repair. Crit. Rev. Biochem. Mol. Biol..

[67] Braun K.A., Lao Y., He Z., Ingles C.J., Wold M.S. (1997). Role of protein-protein interactions in the function of replication protein a (RPA): RPA modulates the activity of DNA polymerase α by multiple mechanisms. Biochemistry.

[68] Mason A.C., Roy R., Simmons D.T., Wold M.S. (2010). Functions of alternative Replication Protein A (aRPA) ininitiation and elongation. Biochemistry.

[69] Haring S.J., Humphreys T.D., Wold M.S. (2009). A naturally occurring human RPA subunit homolog does not support DNA replication or cell-cycle progression. Nucleic Acids Res..

[70] Mason A.C., Haring S.J., Pryor J.M., Staloch C.A., Gan T.F., Wold M.S. (2009). An alternative form of replication protein A prevents viral replication *in vitro*. J. Biol. Chem..

[71] Choi J.-H., Lindsey-Boltz L.A., Kemp M., Mason A.C., Wold M.S., Sancar A. (2010). Reconstitution of RPA-covered single-stranded DNA-activated ATR-Chk1 signaling. PNAS.

[72] Ball H.L., Myers J.S., Cortez D. (2005). ATRIP binding to replication protein A-single-stranded DNA promotes ATR–ATRIP localization but is dispensable for Chk1 phosphorylation. Mol. Biol. Cell.

[73] Zou L., Elledge S.J. (2003). Sensing DNA damage through ATRIP recognition of RPA-ssDNA complexes. Science.

[74] Yang X.H., Zou L. (2006). Recruitment of ATR-ATRIP, Rad17, and 9-1-1 complexes to DNA damage. Methods Enzymol..

[75] Reilly P.T., Yu Y., Hamiche A., Wang L. (2014). Cracking the ANP32 whips: important functions, unequal requirement, and hints at disease implications. Bioessays.

[76] Saavedra F., Rivera C., Rivas E., Merino P., Garrido D., Hernández S. (2017). PP32 and SET/TAF-I proteins regulate the acetylation of newly synthesized histone H4. Nucleic Acids Res..

[77] Utley R.T., Lacoste N., Jobin-Robitaille O., Allard S., Côté J. (2005). Regulation of NuA4 histone acetyltransferase activity in transcription and DNA repair by phosphorylation of histone H4. Mol. Cell. Biol..

[78] Poveda A., Sendra R. (2008). Site specificity of yeast histone acetyltransferase B complex *in vivo*. FEBS J..

[79] Deegan T.D., Mukherjee P.P., Fujisawa R., Rivera C.P., Labib K. (2020). CMG helicase disassembly is controlled by replication fork DNA, replisome components and a ubiquitin threshold. Elife.

[80] Gangwani L. (2006). Deficiency of the zinc finger protein ZPR1 causes defects in transcription and cell cycle progression. J. Biol. Chem..

[81] Kannan A., Jiang X., He L., Ahmad S., Gangwani L. (2020). ZPR1 prevents R-loop accumulation, upregulates SMN2 expression and rescues spinal muscular atrophy. Brain.

[82] Huttlin E.L., Bruckner R.J., Navarrete-perea J., Cannon J.R., Gebreab F., Gygi M.P. (2022). Dual proteome-scale networks reveal cell-specific remodeling of the human interactome. Cell.

[83] Stark C., Breitkreutz B.J., Reguly T., Boucher L., Breitkreutz A., Tyers M. (2006). BioGRID: a general repository for interaction datasets. Nucleic Acids Res..

[84] Cho N.H., Cheveralls K.C., Brunner A.D., Kim K., Michaelis A.C., Raghavan P. (2022). OpenCell: endogenous tagging for the cartography of human cellular organization. Science.

[85] Alabert C., Bukowski-Wills J.C., Lee S.B., Kustatscher G., Nakamura K., De Lima Alves F. (2014). Nascent chromatin capture proteomics determines chromatin dynamics during DNA replication and identifies unknown fork components. Nat. Cell Biol..

[86] Nakamura K., Kustatscher G., Alabert C., Hödl M., Forne I., Völker-Albert M. (2021). Proteome dynamics at broken replication forks reveal a distinct ATM-directed repair response suppressing DNA double-strand break ubiquitination. Mol. Cell.

[87] Dungrawala H., Rose K.L., Bhat K.P., Mohni K.N., Glick G.G., Couch F.B. (2015). The replication checkpoint prevents two types of fork collapse without regulating replisome stability. Mol. Cell.

[88] Sirbu B.M., McDonald W.H., Dungrawala H., Badu-Nkansah A., Kavanaugh G.M., Chen Y. (2013). Identification of proteins at active, stalled, and collapsed replication forks using isolation of proteins on nascent DNA (iPOND) coupled with mass spectrometry. J. Biol. Chem..

[89] Roux K.J., Kim D.I., Burke B., May D.G. (2018). BioID: a screen for protein-protein interactions. Curr. Protoc. Protein Sci..

[90] Ercilla A., Feu S., Aranda S., Llopis A., Brynjólfsdóttir S.H., Sørensen C.S. (2020). Acute hydroxyurea-induced replication blockade results in replisome components disengagement from nascent DNA without causing fork collapse. Cell Mol. Life Sci..

[91] Woodward A.M., Göhler T., Luciani M.G., Oehlmann M., Ge X., Gartner A. (2006). Excess Mcm2-7 license dormant origins of replication that can be used under conditions of replicative stress. J. Cell Biol..

[92] Ge X.Q., Jackson D.A., Blow J.J. (2007). Dormant origins licensed by excess Mcm2-7 are required for human cells to survive replicative stress. Genes Dev..

[93] Shukla A., Bhalla P., Potdar P.K., Jampala P., Bhargava P. (2021). Transcription-dependent enrichment of the yeast FACT complex influences nucleosome dynamics on the RNA polymerase III-transcribed genes. RNA J..

[94] Martin B.J.E., Chruscicki A.T., Howe L.J. (2018). Transcription promotes the interaction of the facilitates chromatin transactions (FACT) complex with nucleosomes in saccharomyces cerevisiae. Genetics.

[95] Elia A.E.H., Wang D.C., Willis N.A., Boardman A.P., Hajdu I., Adeyemi R.O. (2015). RFWD3-Dependent ubiquitination of RPA regulates repair at stalled replication forks. Mol. Cell.

[96] Cortez D. (2016). Preventing replication fork collapse to maintain genome integrity. DNA Repair (Amst).

[97] Bhat K.P., Cortez D. (2018). RPA and RAD51: fork reversal, fork protection, and genome stability. Nat. Struct. Mol. Biol..

[98] Slade D. (2018). Maneuvers on PCNA rings during DNA replication and repair. Genes (Basel).

[99] Fan L., Bi T., Wang L., Xiao W. (2020). DNA-damage tolerance through PCNA ubiquitination and sumoylation. Biochem. J..

[100] Peng Z., Liao Z., Matsumoto Y., Yang A., Tomkinson A.E. (2016). Human DNA ligase I interacts with and is targeted for degradation by the DCAF7 specificity factor of the Cul4-DDB1 ubiquitin ligase complex. J. Biol. Chem..

[101] Sogo J.M., Lopes M., Foiani M. (2002). Fork reversal and ssDNA accumulation at stalled replication forks owing to checkpoint defects. Science.

[102] Petermann E., Orta M.L., Issaeva N., Schultz N., Helleday T. (2010). Supplemental information - hydroxyurea-stalled replication forks become progressively inactivated and require two different RAD51-mediated pathways for restart and repair. Mol. Cell.

[103] Yuan Y., Stumpf F.M., Schlor L.A., Schmidt O.P., Saumer P., Huber L.B. (2023). Chemoproteomic discovery of a human RNA ligase. Nat. Commun..

[104] Jones R.M., Melton P.E., Pinese M., Rea A.J., Ingley E., Ballinger M.L. (2019). Identification of novel sarcoma risk genes using a two-stage genome wide DNA sequencing strategy in cancer cluster families and population case and control cohorts. BMC Med. Genet..

[105] Fodor E., Velthuis A.J.W.T. (2020). Structure and function of the influenza virus transcription and replication machinery. Cold Spring Harb. Perspect. Med..

[106] Lopez-Contreras A.J., Ruppen I., Nieto-Soler M., Murga M., Rodriguez-Acebes S., Remeseiro S. (2013). A proteomic characterization of factors enriched at nascent DNA molecules. Cell Rep..

[107] Olivieri M., Cho T., Álvarez-quilón A., Li K., Matthew J., Zimmermann M. (2021). A genetic map of the response to DNA damage in human cells. Cell.

[108] Perez-Riverol Y., Bai J., Bandla C., García-Seisdedos D., Hewapathirana S., Kamatchinathan S. (2022). The PRIDE database resources in 2022: a hub for mass spectrometry-based proteomics evidences. Nucleic Acids Res..

