# Supplementary material for: Replisome Proximal Protein Associations and Dynamic Proteomic Changes at Stalled Replication Forks
Source: Mol Cell Proteomics. 2024 Apr 13;23(5):100767. doi: 10.1016/j.mcpro.2024.100767 (PMC11101681; doi:10.1016/j.mcpro.2024.100767)
Supplement: Supplemental Figure S1 — Hydroxyurea-induced DNA damage in 293FT cells and bait protein expression. A, 293FT cells were treated with increasing hydroxyurea concentrations for 24 h. The extent of DNA damage was analyzed by immunoblotting using a γ-H2AX antibody. B, Immunolabeling of HU-treated HEK293 Flp-In T-REx (293FT) cells showing γ-H2AX accumulation 24 hrs after treatment. Scale bars = 10 μm. C, Flow cytometry analysis of propidium iodine-stained cells with or without exposure to 1 mM hydroxyurea for 24 h (n = 3). D, Quantification of the cell proportion in each phase of the cell cycle. The experiments were independently repeated three times, and the data was presented by mean ± standard deviation (SD). Statistical analyses were done using a two-way ANOVA test, with p < 0.05 considered as significantly different (ns = non-significant, ∗∗p < 0.001). E–U, Expression of the different BioID2 fusion proteins verified by immunoblotting. Isogenic 293FT cells expressing each of the bait proteins were induced with doxycycline for 48 h and detected using Myc or Flag epitope tags. The γH2AX marker was used as a surrogate for DNA damage in cells exposed to 1 mM Hydroxyurea for 24 h. The expressed bait protein is indicated over each set of blots. Supplemental Figure S1: Immunofluorescent labeling of bait proteins showing their sub-cellular localization. Isogenic HEK293 Flp-In T-REx cells were induced with doxycycline for 48 h and bait proteins detected using antibodies against either Myc or Flag tags. The expressed bait protein is indicated over each set of micrographs. Scale bars = 10 μm. Supplemental Figure S3: Scatter plots showing the proximal associations for RPA proteins in untreated cells. Prey proteins were considered enriched over the 293FT control if the SAINT score ≥0.95. The colored dots further denote prey proteins with a fold change enrichment ≥1.5 in over the GFP and GFP-NLS controls. Labeled proteins represent nuclear prey proteins identified using at least two negative controls. Dat [file mmc18.pdf]

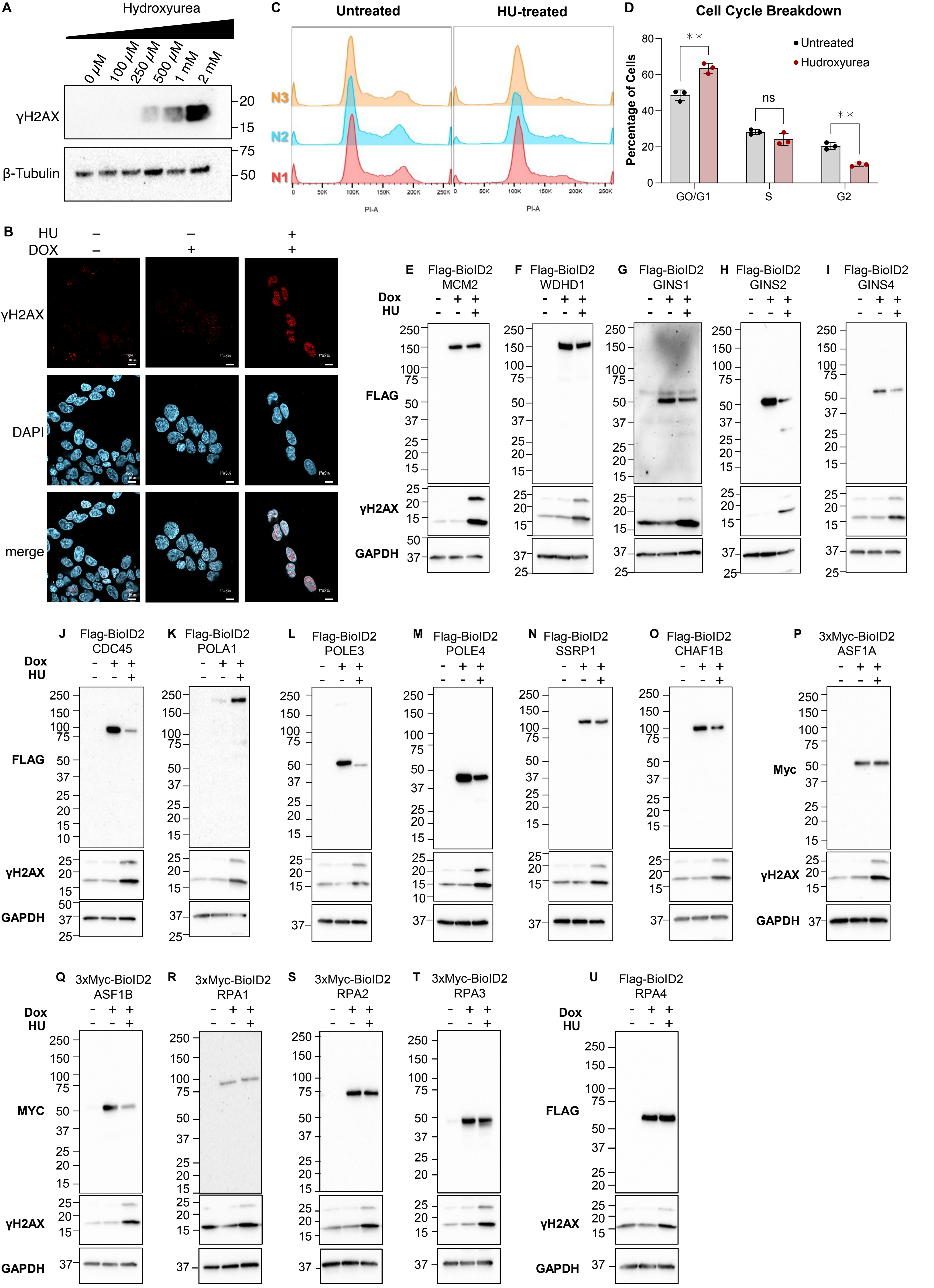

A
Hydroxyurea
0 μM
100 μM
250 μM
500 μM
1 mM
2 mM
γH2AX
β-Tubulin
C
Untreated
HU-treated
N3
N2
N1
PI-A
D
Cell Cycle Breakdown
Percentage of Cells
Untreated
Hudroxyurea
GO/G1
S
G2
ns
B
HU
DOX
γH2AX
DAPI
merge
E Flag-BioID2 MCM2
F Flag-BioID2 WDHD1
G Flag-BioID2 GINS1
H Flag-BioID2 GINS2
I Flag-BioID2 GINS4
Dox
HU
FLAG
γH2AX
GAPDH
J Flag-BioID2 CDC45
K Flag-BioID2 POLA1
L Flag-BioID2 POLE3
M Flag-BioID2 POLE4
N Flag-BioID2 SSRP1
O Flag-BioID2 CHAF1B
P 3xMyc-BioID2 ASF1A
Myc
Q 3xMyc-BioID2 ASF1B
R 3xMyc-BioID2 RPA1
S 3xMyc-BioID2 RPA2
T 3xMyc-BioID2 RPA3
U Flag-BioID2 RPA4
MYC

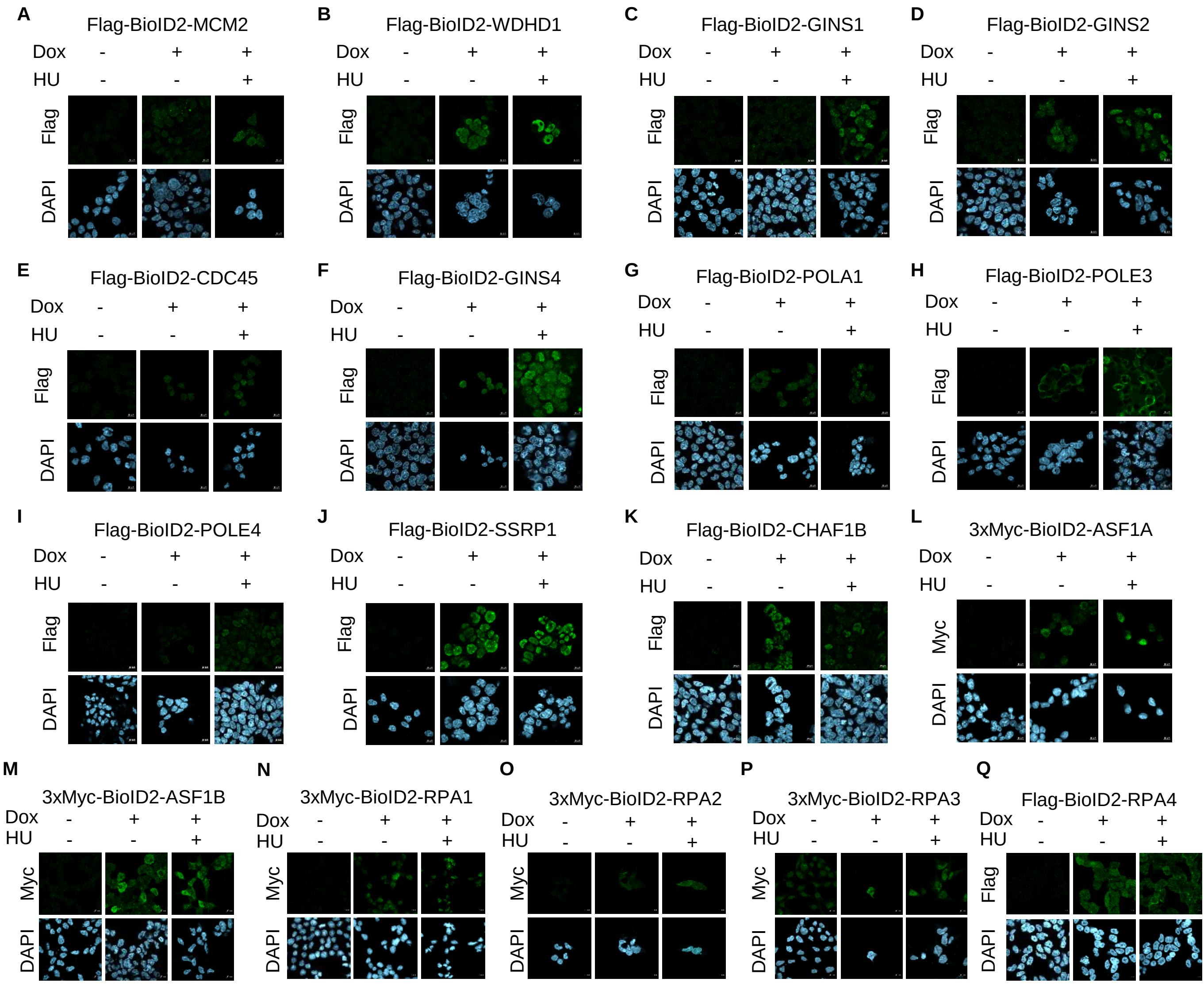

A
Flag-BioID2-MCM2
B
Flag-BioID2-WDHD1
C
Flag-BioID2-GINS1
D
Flag-BioID2-GINS2
E
Flag-BioID2-CDC45
F
Flag-BioID2-GINS4
G
Flag-BioID2-POLA1
H
Flag-BioID2-POLE3
I
Flag-BioID2-POLE4
J
Flag-BioID2-SSRP1
K
Flag-BioID2-CHAF1B
L
3xMyc-BioID2-ASF1A
M
3xMyc-BioID2-ASF1B
N
3xMyc-BioID2-RPA1
O
3xMyc-BioID2-RPA2
P
3xMyc-BioID2-RPA3
Q
Flag-BioID2-RPA4
Dox - + +
HU - - +
Flag
Myc
DAPI

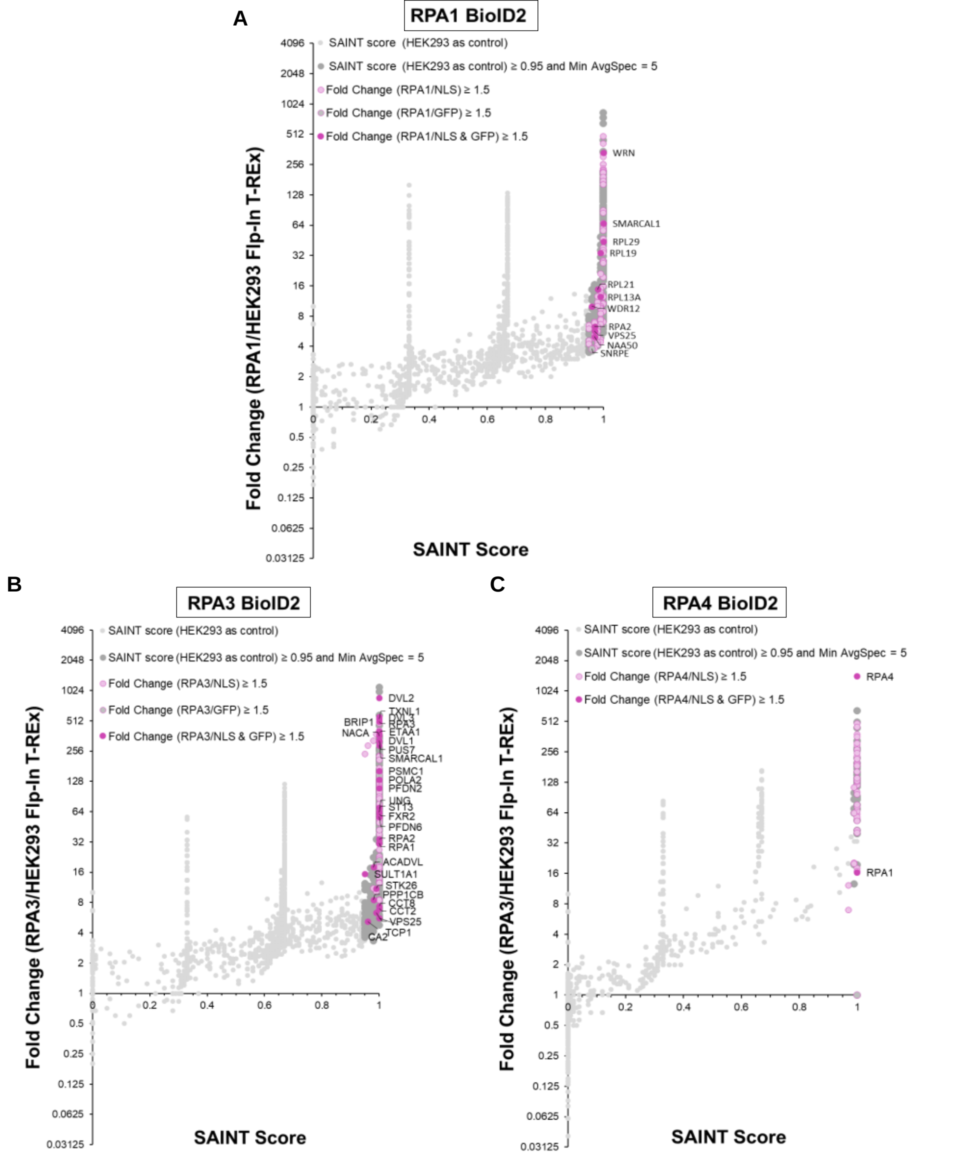
A
RPA1 BioID2
SAINT score (HEK293 as control)
SAINT score (HEK293 as control) ≥ 0.95 and Min AvgSpec = 5
Fold Change (RPA1/NLS) ≥ 1.5
Fold Change (RPA1/GFP) ≥ 1.5
Fold Change (RPA1/NLS & GFP) ≥ 1.5
WRN
SMARCAL1
RPL29
RPL19
RPL21
RPL13A
WDR12
RPA2
VPS25
NAA50
SNRPE
Fold Change (RPA1/HEK293 Flp-In T-REx)
SAINT Score
B
RPA3 BioID2
SAINT score (HEK293 as control)
SAINT score (HEK293 as control) ≥ 0.95 and Min AvgSpec = 5
Fold Change (RPA3/NLS) ≥ 1.5
Fold Change (RPA3/GFP) ≥ 1.5
Fold Change (RPA3/NLS & GFP) ≥ 1.5
DVL2
TXNL1
DVL3
BRIP1
RPA3
NACA
ETAA1
DVL1
PUS7
SMARCAL1
PSMC1
POLA2
PFDN2
UNG
STI3
FXR2
PFDN6
RPA2
RPA1
ACADVL
SULT1A1
STK26
PPP1CB
CCT8
CCT2
VPS25
TCP1
CA2
Fold Change (RPA3/HEK293 Flp-In T-REx)
SAINT Score
C
RPA4 BioID2
SAINT score (HEK293 as control)
SAINT score (HEK293 as control) ≥ 0.95 and Min AvgSpec = 5
Fold Change (RPA4/NLS) ≥ 1.5
Fold Change (RPA4/NLS & GFP) ≥ 1.5
RPA4
RPA1
Fold Change (RPA3/HEK293 Flp-In T-REx)
SAINT Score

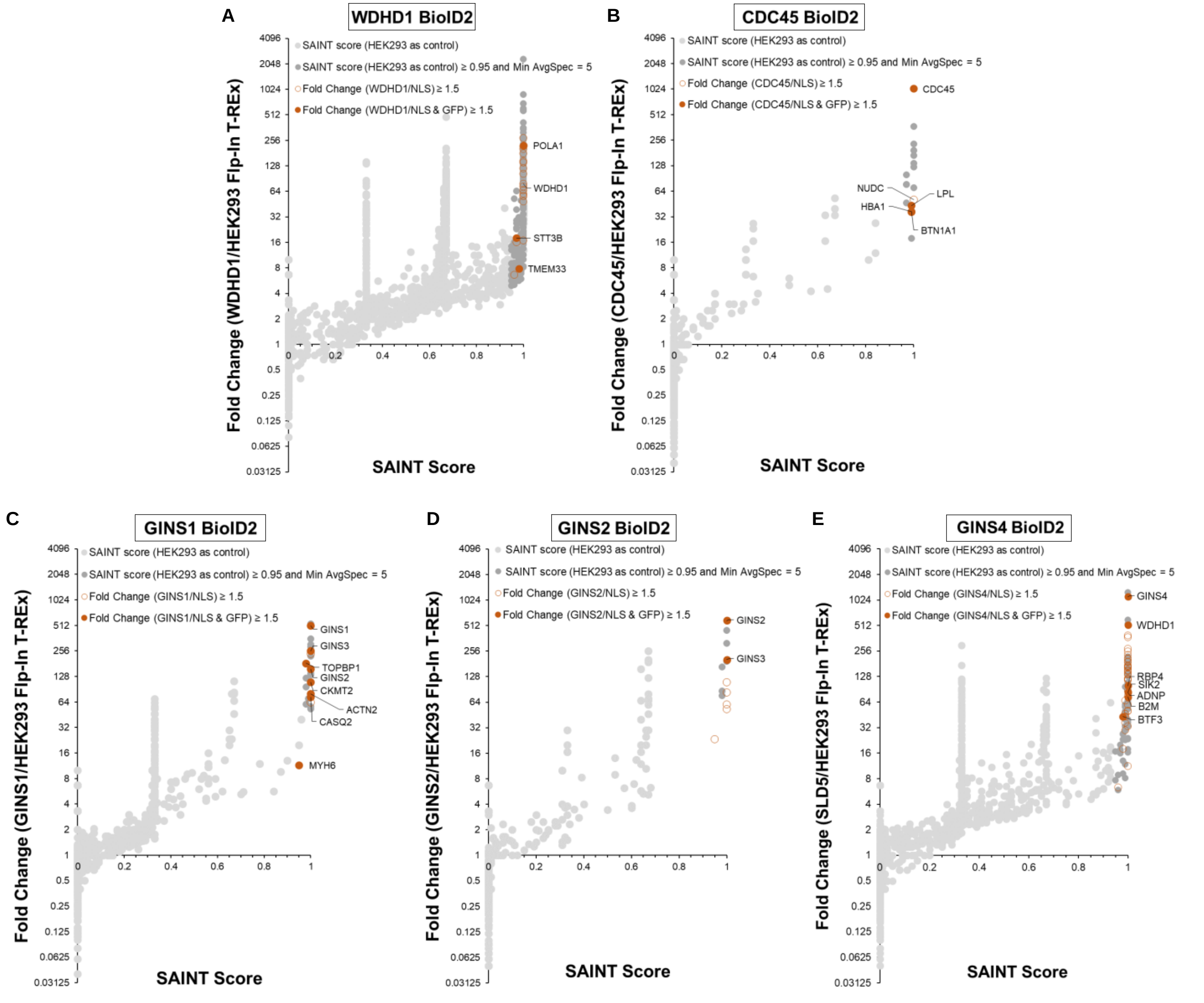
A
WDHD1 BioID2
SAINT score (HEK293 as control)
SAINT score (HEK293 as control) ≥ 0.95 and Min AvgSpec = 5
Fold Change (WDHD1/NLS) ≥ 1.5
Fold Change (WDHD1/NLS & GFP) ≥ 1.5
POLA1
WDHD1
STT3B
TMEM33
Fold Change (WDHD1/HEK293 Flp-In T-REx)
SAINT Score
B
CDC45 BioID2
SAINT score (HEK293 as control)
SAINT score (HEK293 as control) ≥ 0.95 and Min AvgSpec = 5
Fold Change (CDC45/NLS) ≥ 1.5
Fold Change (CDC45/NLS & GFP) ≥ 1.5
CDC45
NUDC
LPL
HBA1
BTN1A1
Fold Change (CDC45/HEK293 Flp-In T-REx)
SAINT Score
C
GINS1 BioID2
SAINT score (HEK293 as control)
SAINT score (HEK293 as control) ≥ 0.95 and Min AvgSpec = 5
Fold Change (GINS1/NLS) ≥ 1.5
Fold Change (GINS1/NLS & GFP) ≥ 1.5
GINS1
GINS3
TOPBP1
GINS2
CKMT2
ACTN2
CASQ2
MYH6
Fold Change (GINS1/HEK293 Flp-In T-REx)
SAINT Score
D
GINS2 BioID2
SAINT score (HEK293 as control)
SAINT score (HEK293 as control) ≥ 0.95 and Min AvgSpec = 5
Fold Change (GINS2/NLS) ≥ 1.5
Fold Change (GINS2/NLS & GFP) ≥ 1.5
GINS2
GINS3
Fold Change (GINS2/HEK293 Flp-In T-REx)
SAINT Score
E
GINS4 BioID2
SAINT score (HEK293 as control)
SAINT score (HEK293 as control) ≥ 0.95 and Min AvgSpec = 5
Fold Change (GINS4/NLS) ≥ 1.5
Fold Change (GINS4/NLS & GFP) ≥ 1.5
GINS4
WDHD1
RBP4
SIK2
ADNP
B2M
BTF3
Fold Change (SLD5/HEK293 Flp-In T-REx)
SAINT Score

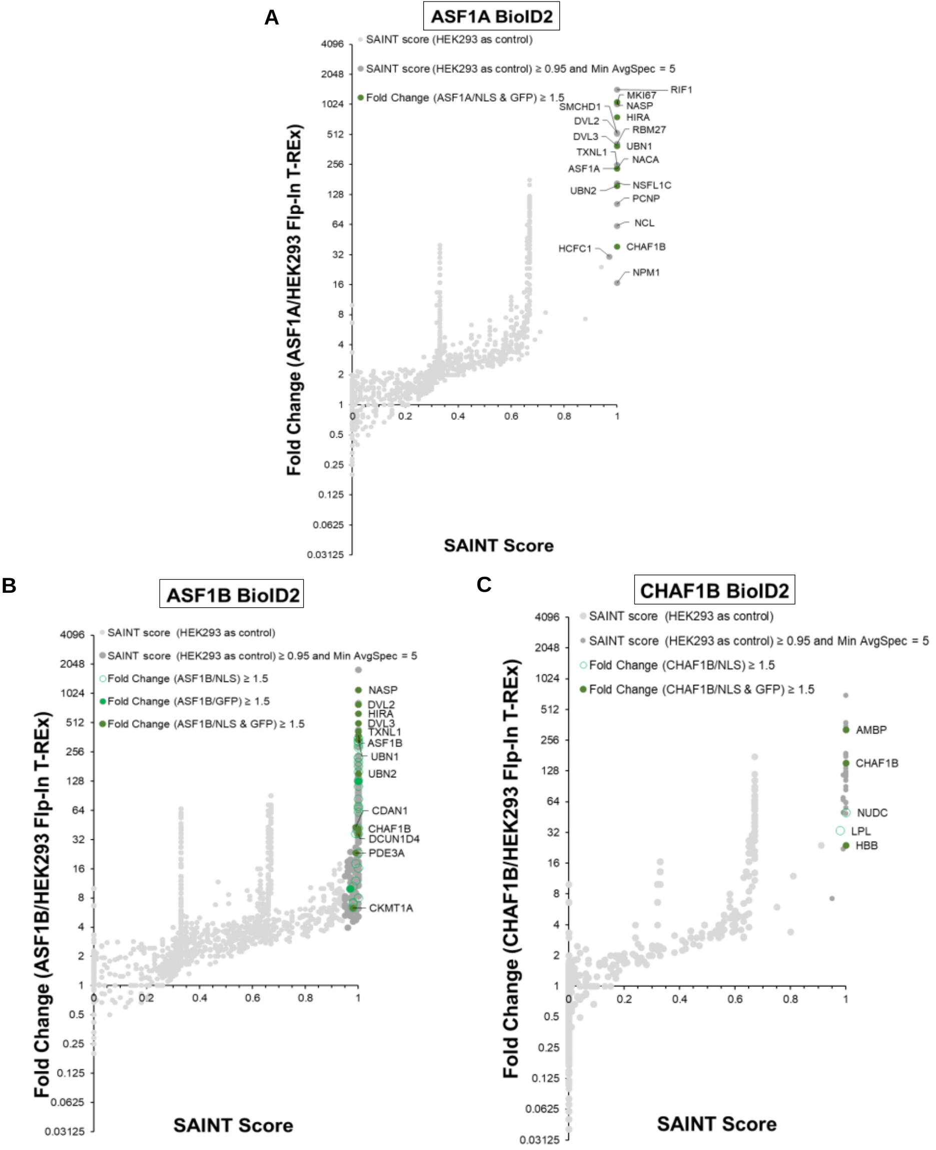

A
ASF1A BioID2
SAINT score (HEK293 as control)
SAINT score (HEK293 as control) ≥ 0.95 and Min AvgSpec = 5
Fold Change (ASF1A/NLS & GFP) ≥ 1.5
RIF1
MKI67
NASP
SMCHD1
HIRA
DVL2
RBM27
DVL3
UBN1
TXNL1
NACA
ASF1A
NSFL1C
UBN2
PCNP
NCL
CHAF1B
HCFC1
NPM1
Fold Change (ASF1A/HEK293 Flp-In T-REx)
SAINT Score
B
ASF1B BioID2
SAINT score (HEK293 as control)
SAINT score (HEK293 as control) ≥ 0.95 and Min AvgSpec = 5
Fold Change (ASF1B/NLS) ≥ 1.5
Fold Change (ASF1B/GFP) ≥ 1.5
Fold Change (ASF1B/NLS & GFP) ≥ 1.5
NASP
DVL2
HIRA
DVL3
TXNL1
ASF1B
UBN1
UBN2
CDAN1
CHAF1B
DCUN1D4
PDE3A
CKMT1A
Fold Change (ASF1B/HEK293 Flp-In T-REx)
SAINT Score
C
CHAF1B BioID2
SAINT score (HEK293 as control)
SAINT score (HEK293 as control) ≥ 0.95 and Min AvgSpec = 5
Fold Change (CHAF1B/NLS) ≥ 1.5
Fold Change (CHAF1B/NLS & GFP) ≥ 1.5
AMBP
CHAF1B
NUDC
LPL
HBB
Fold Change (CHAF1B/HEK293 Flp-In T-REx)
SAINT Score

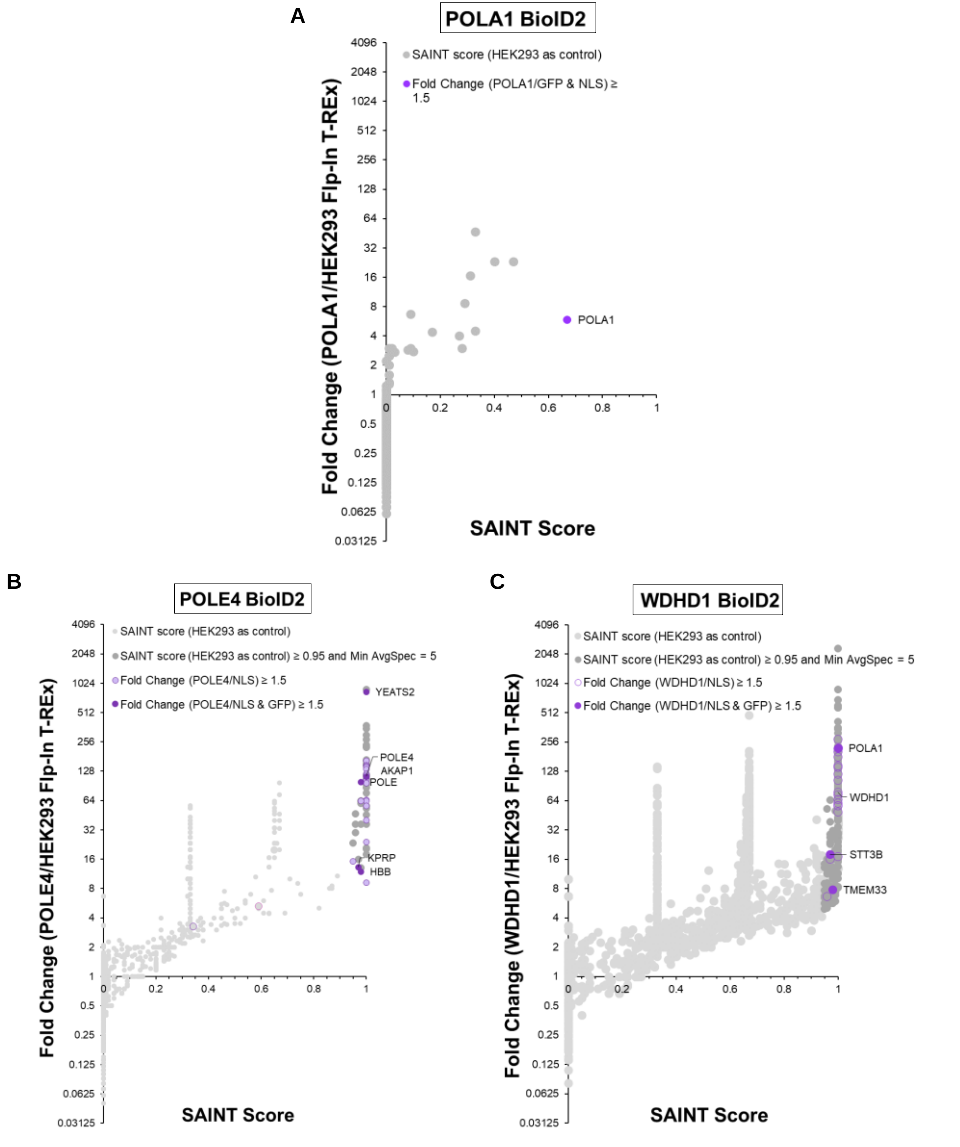
A
POLA1 BioID2
SAINT score (HEK293 as control)
Fold Change (POLA1/GFP & NLS) ≥ 1.5
POLA1
Fold Change (POLA1/HEK293 Flp-In T-REx)
SAINT Score
B
POLE4 BioID2
SAINT score (HEK293 as control)
SAINT score (HEK293 as control) ≥ 0.95 and Min AvgSpec = 5
Fold Change (POLE4/NLS) ≥ 1.5
Fold Change (POLE4/NLS & GFP) ≥ 1.5
YEATS2
POLE4
AKAP1
POLE
KPRP
HBB
Fold Change (POLE4/HEK293 Flp-In T-REx)
SAINT Score
C
WDHD1 BioID2
SAINT score (HEK293 as control)
SAINT score (HEK293 as control) ≥ 0.95 and Min AvgSpec = 5
Fold Change (WDHD1/NLS) ≥ 1.5
Fold Change (WDHD1/NLS & GFP) ≥ 1.5
POLA1
WDHD1
STT3B
TMEM33
Fold Change (WDHD1/HEK293 Flp-In T-REx)
SAINT Score

**A**

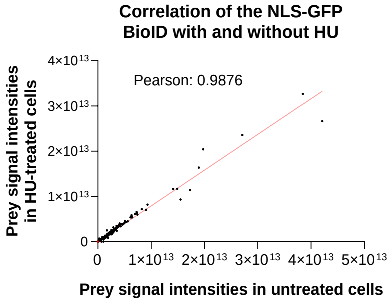


**B**

| BioID ASF1A | Unique peptides | Sequence Coverage (%) |
|---|---|---|
| ASF1A | 7 | 48 |
| ASF1B | 3 | 33,7 |
| CHAF1B | 30 | 58,5 |
| SSRP1 | 28 | 44,9 |

| BioID ASF1B | Unique peptides | Sequence Coverage (%) |
|---|---|---|
| ASF1A | - | - |
| ASF1B | 10 | 88,9 |
| CHAF1B | 26 | 56 |
| SSRP1 | 29 | 46 |

| BioID MCM2 | Unique peptides | Sequence Coverage (%) |
|---|---|---|
| MCM2 | 75 | 75,2 |
| GINS1 | 3 | 18,9 |
| GINS2 | - | - |
| GINS4 | 5 | 30,5 |
| CDC45 | - | - |
| CTF4 | 46 | 53 |

| BioID CTF4 | Unique peptides | Sequence Coverage (%) |
|---|---|---|
| MCM2 | 31 | 44,2 |
| GINS1 | - | - |
| GINS2 | - | - |
| GINS4 | 5 | 30,5 |
| CDC45 | - | - |
| CTF4 | 110 | 81,8 |

| BioID SSRP1 | Unique peptides | Sequence Coverage (%) |
|---|---|---|
| ASF1A | - | - |
| ASF1B | 4 | 33,7 |
| CHAF1B | 14 | 29,9 |
| SSRP1 | 68 | 71,7 |

| BioID CHAF1B | Unique peptides | Sequence Coverage (%) |
|---|---|---|
| ASF1A | - | - |
| ASF1B | 4 | 33,7 |
| CHAF1B | 46 | 71,9 |
| SSRP1 | 28 | 45,8 |

| BioID GINS1 | Unique peptides | Sequence Coverage (%) |
|---|---|---|
| MCM2 | 35 | 47,7 |
| GINS1 | 25 | 83,2 |
| GINS2 | 11 | 67 |
| GINS4 | 5 | 30,5 |
| CDC45 | - | - |
| CTF4 | 46 | 52,8 |

| BioID GINS2 | Unique peptides | Sequence Coverage (%) |
|---|---|---|
| MCM2 | 31 | 44,2 |
| GINS1 | 13 | 64,3 |
| GINS2 | 18 | 87,6 |
| GINS4 | 16 | 70,4 |
| CDC45 | - | - |
| CTF4 | 46 | 53 |

| BioID RPA1 | Unique peptides | Sequence Coverage (%) |
|---|---|---|
| RPA1 | 63 | 89,1 |
| RPA2 | 10 | 60 |
| RPA3 | - | - |
| RPA4 | - | - |

| BioID RPA2 | Unique peptides | Sequence Coverage (%) |
|---|---|---|
| RPA1 | 48 | 84,9 |
| RPA2 | 27 | 97,4 |
| RPA3 | 3 | 37,2 |
| RPA4 | - | - |

| BioID GINS4 | Unique peptides | Sequence Coverage (%) |
|---|---|---|
| MCM2 | 31 | 44,2 |
| GINS1 | 4 | 27,6 |
| GINS2 | - | - |
| GINS4 | 24 | 78 |
| CDC45 | - | - |
| CTF4 | 52 | 57,4 |

| BioID CDC45 | Unique peptides | Sequence Coverage (%) |
|---|---|---|
| MCM2 | 33 | 46,1 |
| GINS1 | - | - |
| GINS2 | - | - |
| GINS4 | 5 | 30,5 |
| CDC45 | 45 | 65,7 |
| CTF4 | 65 | 63,7 |

| BioID RPA3 | Unique peptides | Sequence Coverage (%) |
|---|---|---|
| RPA1 | 54 | 86,4 |
| RPA2 | 16 | 88,5 |
| RPA3 | 6 | 100 |
| RPA4 | - | - |

| BioID RPA4 | Unique peptides | Sequence Coverage (%) |
|---|---|---|
| RPA1 | 40 | 78,9 |
| RPA2 | 3 | 15,6 |
| RPA3 | 6 | 100 |
| RPA4 | 39 | 97,7 |

| BioID POLE4 | Unique peptides | Sequence Coverage (%) |
|---|---|---|
| POLA1 | 43 | 41,2 |
| POLE3 | 3 | 27,9 |
| POLE4 | 5 | 51,3 |
| CTF4 | 46 | 52,8 |

| BioID CTF4 | Unique peptides | Sequence Coverage (%) |
|---|---|---|
| POLA1 | 64 | 53 |
| POLE3 | - | - |
| POLE4 | - | - |
| CTF4 | 110 | 81,8 |

| BioID POLA1 | Unique peptides | Sequence Coverage (%) |
|---|---|---|
| POLA1 | 95 | 65,3 |
| POLE3 | - | - |
| POLE4 | - | - |
| CTF4 | 47 | 53,7 |

| BioID POLA1 | Unique peptides | Sequence Coverage (%) |
|---|---|---|
| POLA1 | 95 | 65,3 |
| POLE3 | - | - |
| POLE4 | - | - |
| CTF4 | 47 | 53,7 |

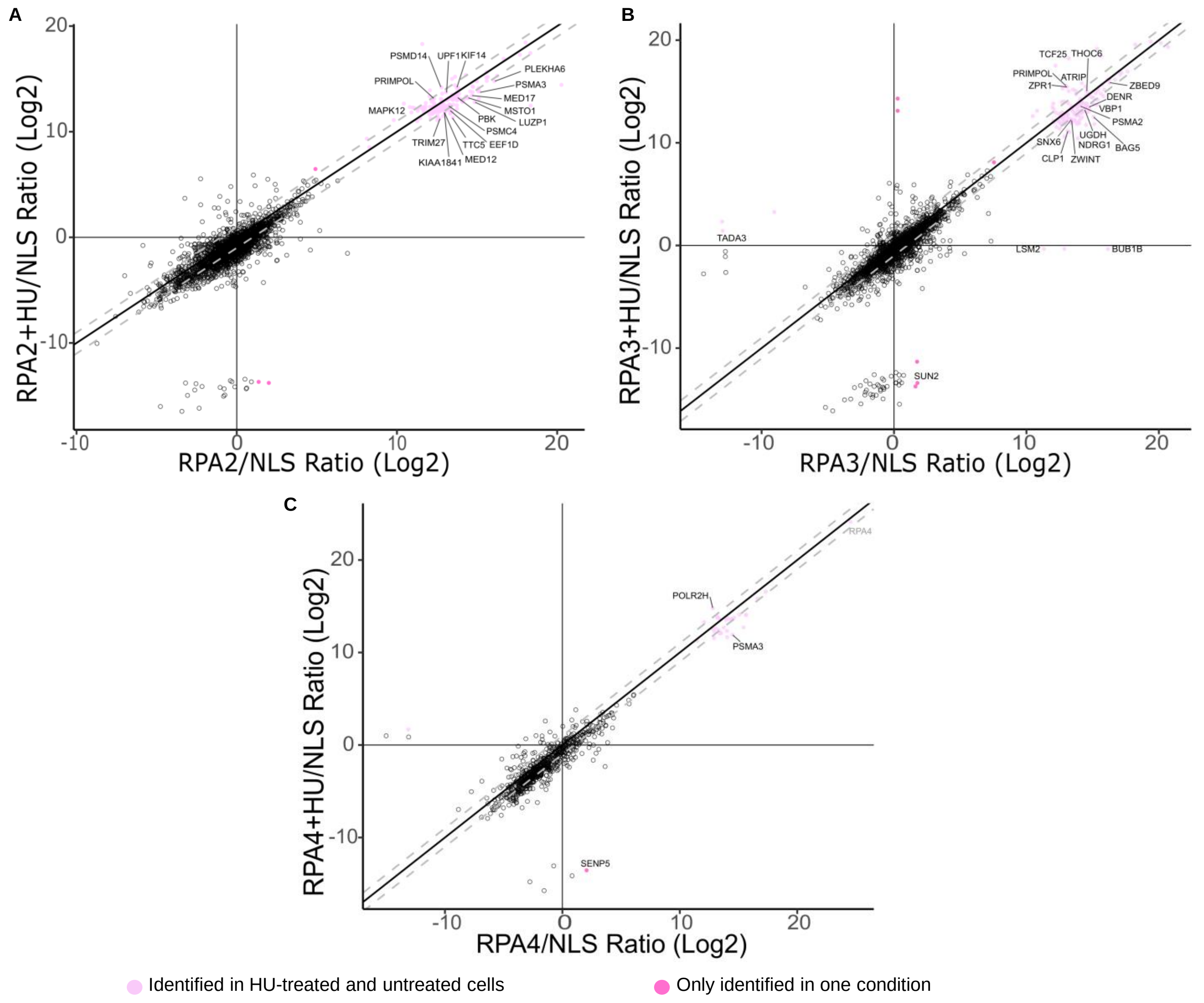
A
RPA2+HU/NLS Ratio (Log2)
RPA2/NLS Ratio (Log2)
20
10
0
-10
-10
0
10
20
PSMD14
UPF1
KIF14
PLEKHA6
PRIMPOL
PSMA3
MED17
MAPK12
MSTO1
LUZP1
PBK
PSMC4
TRIM27
TTC5
EEF1D
KIAA1841
MED12
B
RPA3+HU/NLS Ratio (Log2)
RPA3/NLS Ratio (Log2)
TCF25
THOC6
PRIMPOL
ATRIP
ZPR1
ZBED9
DENR
VBP1
PSMA2
UGDH
SNX6
NDRG1
BAG5
CLP1
ZWINT
TADA3
LSM2
BUB1B
SUN2
C
RPA4+HU/NLS Ratio (Log2)
RPA4/NLS Ratio (Log2)
RPA4
POLR2H
PSMA3
SENP5
Identified in HU-treated and untreated cells
Only identified in one condition

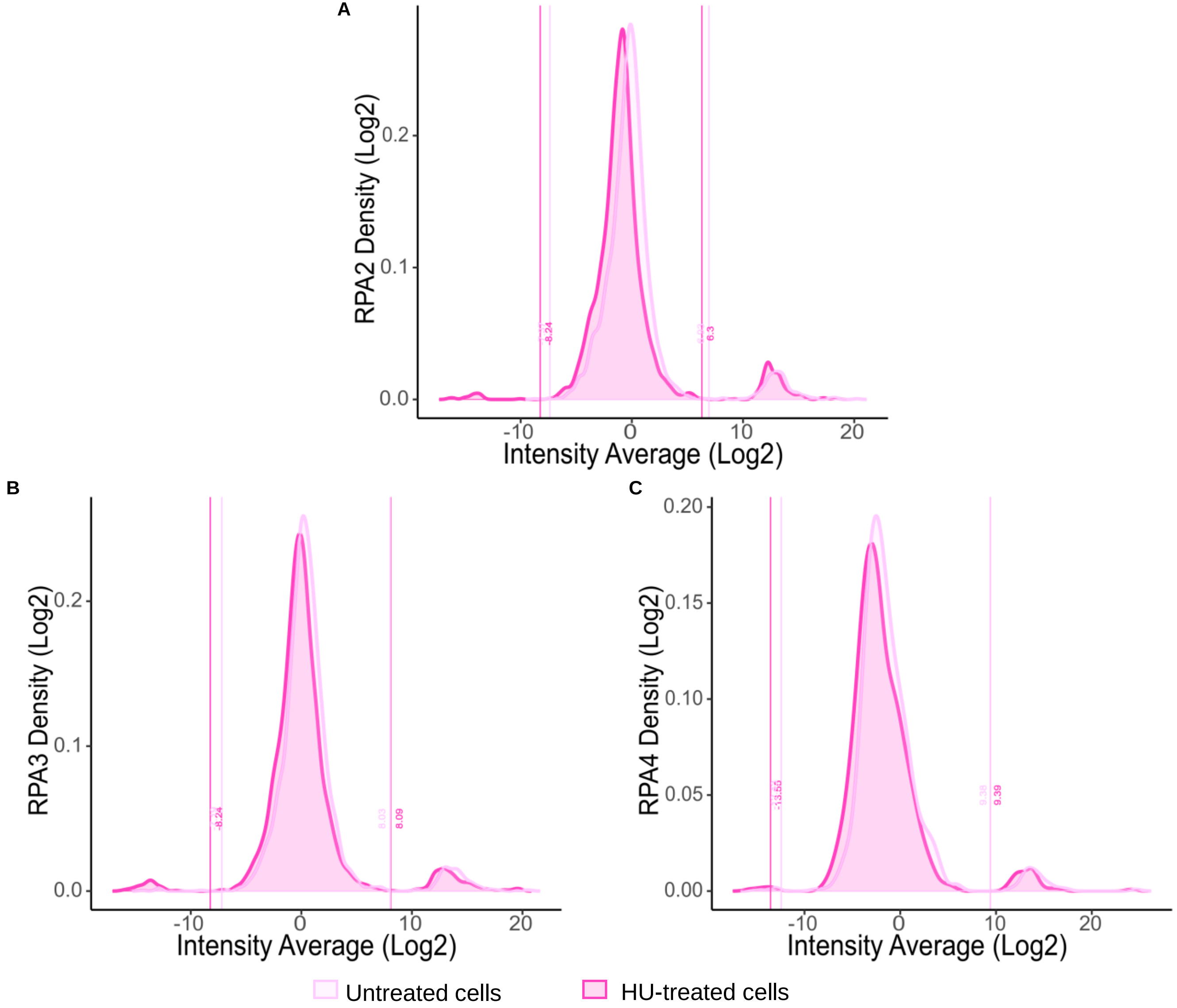
A
RPA2 Density (Log2)
0.0
0.1
0.2
-10
0
10
20
Intensity Average (Log2)
-8.24
6.3
B
RPA3 Density (Log2)
0.0
0.1
0.2
-10
0
10
20
Intensity Average (Log2)
-8.24
8.03
8.09
C
RPA4 Density (Log2)
0.00
0.05
0.10
0.15
0.20
-10
0
10
20
Intensity Average (Log2)
-13.55
9.38
9.39
Untreated cells
HU-treated cells

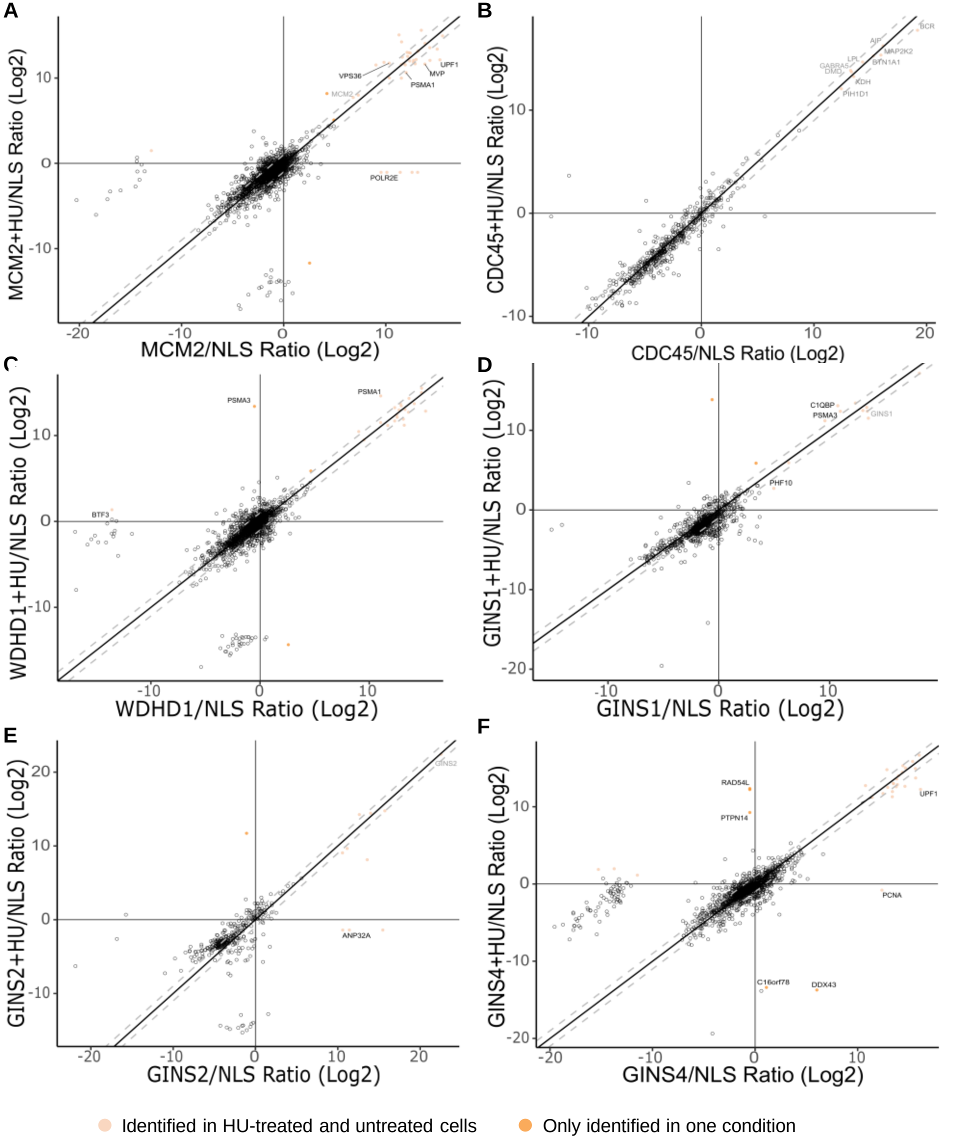
A
MCM2+HU/NLS Ratio (Log2)
MCM2/NLS Ratio (Log2)
VPS36
UPF1
MVP
PSMA1
MCM2
POLR2E
B
CDC45+HU/NLS Ratio (Log2)
CDC45/NLS Ratio (Log2)
BCR
AIP
MAP2K2
LPL
BTN1A1
GABRA5
DMD
XDH
PIH1D1
C
WDHD1+HU/NLS Ratio (Log2)
WDHD1/NLS Ratio (Log2)
PSMA3
PSMA1
BTF3
D
GINS1+HU/NLS Ratio (Log2)
GINS1/NLS Ratio (Log2)
C1QBP
PSMA3
GINS1
PHF10
E
GINS2+HU/NLS Ratio (Log2)
GINS2/NLS Ratio (Log2)
GINS2
ANP32A
F
GINS4+HU/NLS Ratio (Log2)
GINS4/NLS Ratio (Log2)
RAD54L
PTPN14
UPF1
PCNA
C16orf78
DDX43
Identified in HU-treated and untreated cells
Only identified in one condition

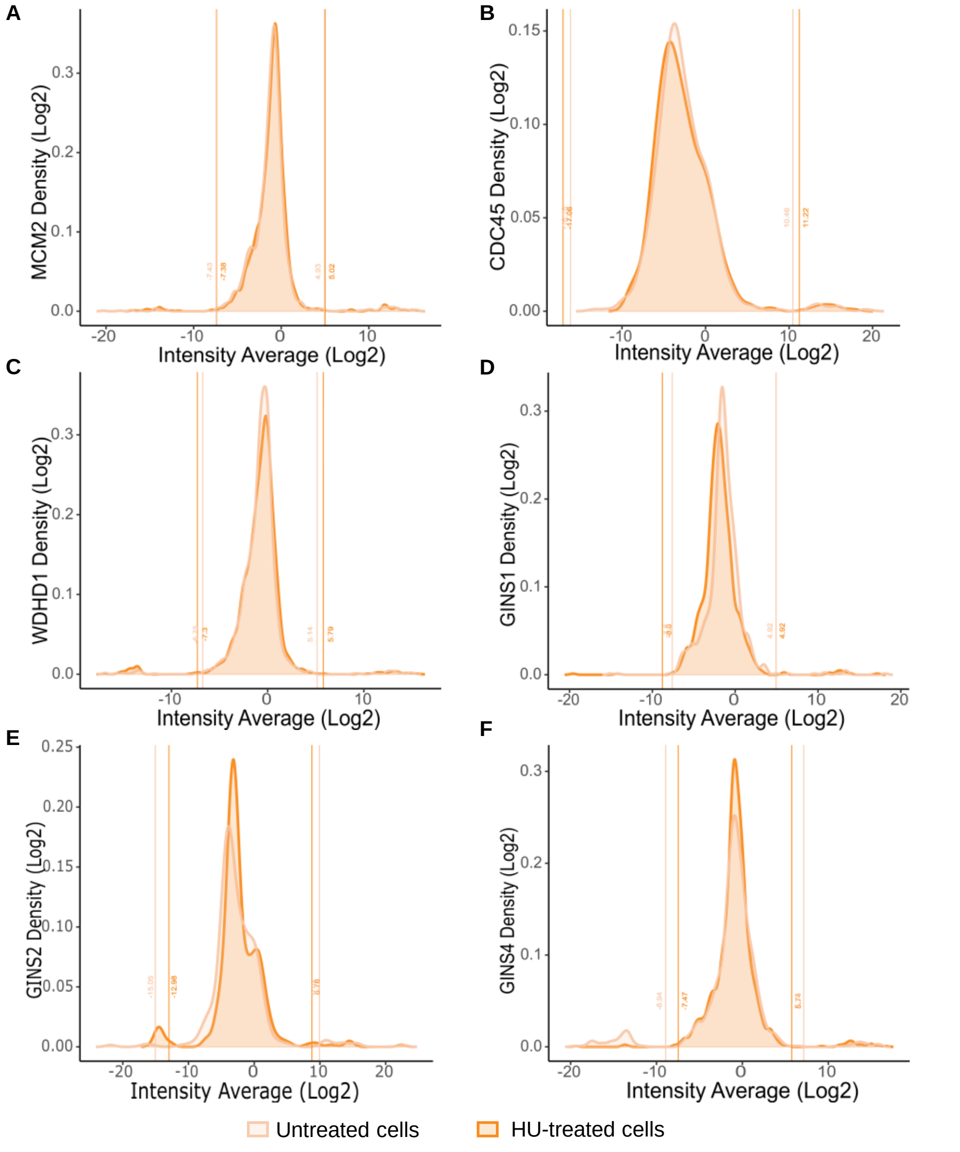
A
MCM2 Density (Log2)
Intensity Average (Log2)
B
CDC45 Density (Log2)
Intensity Average (Log2)
C
WDHD1 Density (Log2)
Intensity Average (Log2)
D
GINS1 Density (Log2)
Intensity Average (Log2)
E
GINS2 Density (Log2)
Intensity Average (Log2)
F
GINS4 Density (Log2)
Intensity Average (Log2)
Untreated cells
HU-treated cells

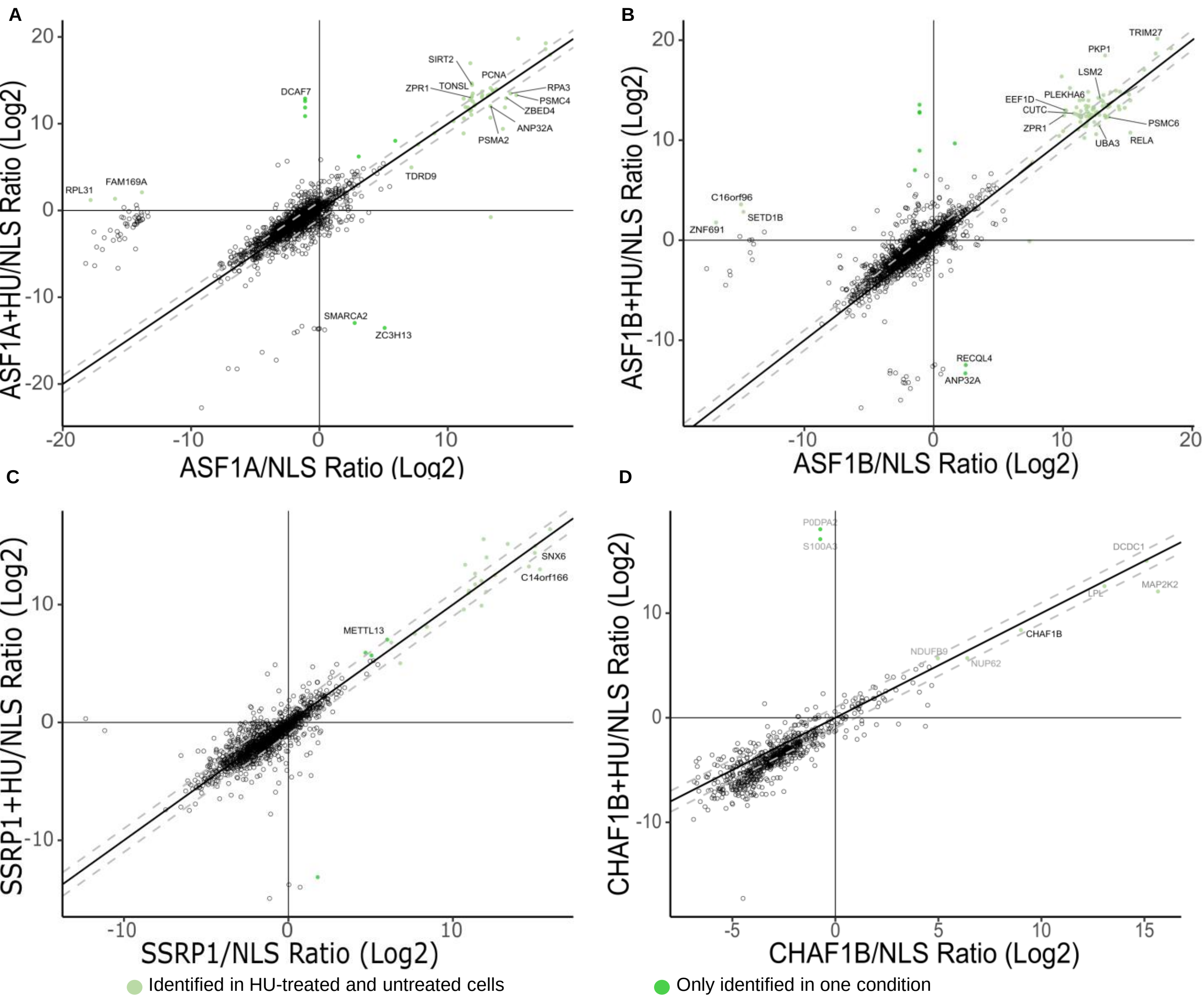
A
B
C
D
ASF1A/NLS Ratio (Log2)
ASF1A+HU/NLS Ratio (Log2)
ASF1B/NLS Ratio (Log2)
ASF1B+HU/NLS Ratio (Log2)
SSRP1/NLS Ratio (Log2)
SSRP1+HU/NLS Ratio (Log2)
CHAF1B/NLS Ratio (Log2)
CHAF1B+HU/NLS Ratio (Log2)
SIRT2
PCNA
DCAF7
ZPR1
TONSL
RPA3
PSMC4
ZBED4
ANP32A
PSMA2
TDRD9
RPL31
FAM169A
SMARCA2
ZC3H13
TRIM27
PKP1
LSM2
PLEKHA6
EEF1D
CUTC
ZPR1
PSMC6
UBA3
RELA
C16orf96
SETD1B
ZNF691
RECQL4
ANP32A
SNX6
C14orf166
METTL13
P0DPA2
S100A3
DCDC1
MAP2K2
LPL
CHAF1B
NDUFB9
NUP62
Identified in HU-treated and untreated cells
Only identified in one condition

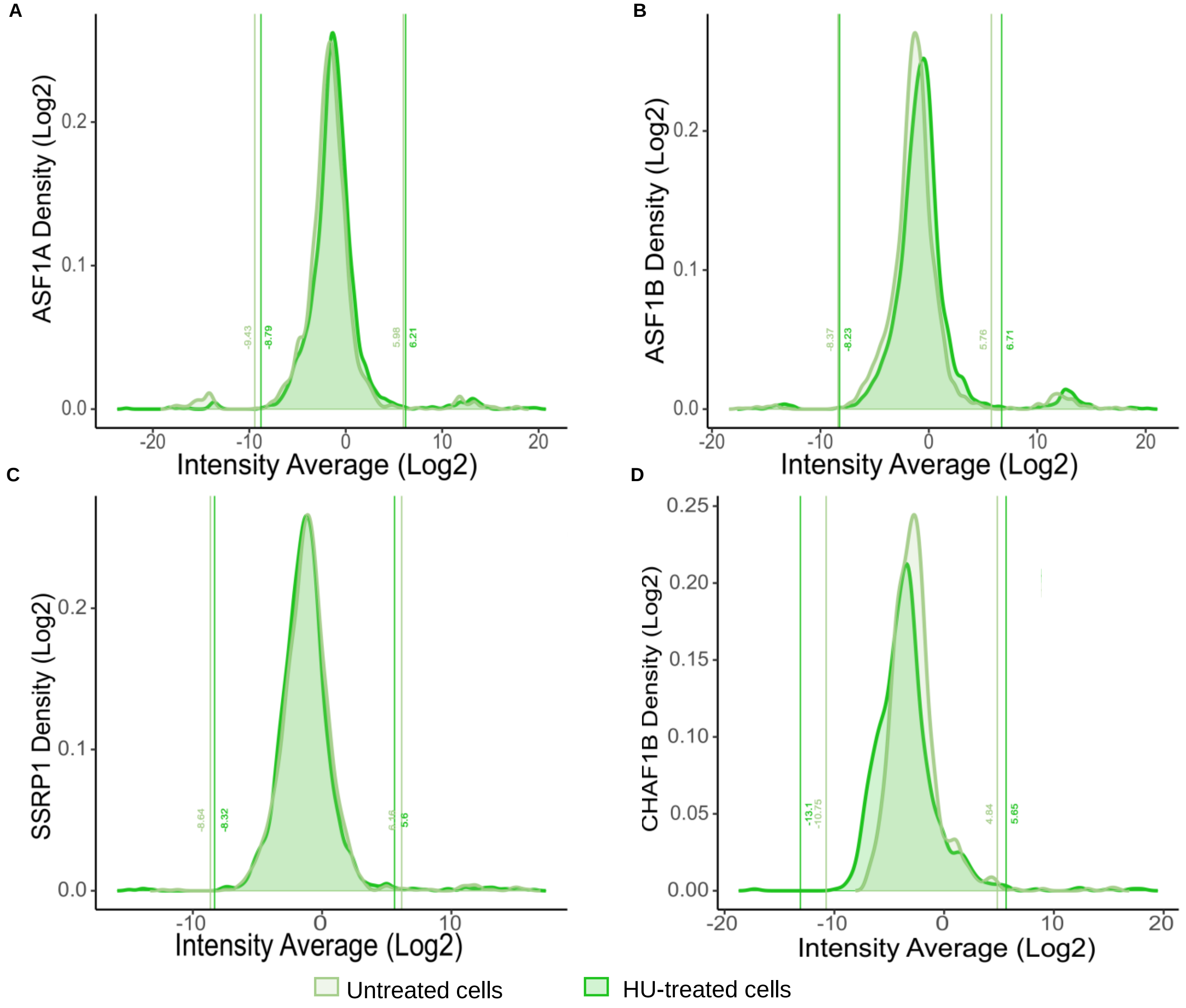

A
ASF1A Density (Log2)
Intensity Average (Log2)
-9.43
-8.79
5.98
6.21
B
ASF1B Density (Log2)
Intensity Average (Log2)
-8.37
-8.23
5.76
6.71
C
SSRP1 Density (Log2)
Intensity Average (Log2)
-8.64
-8.32
6.16
5.6
D
CHAF1B Density (Log2)
Intensity Average (Log2)
-13.1
-10.75
4.84
5.65
Untreated cells
HU-treated cells

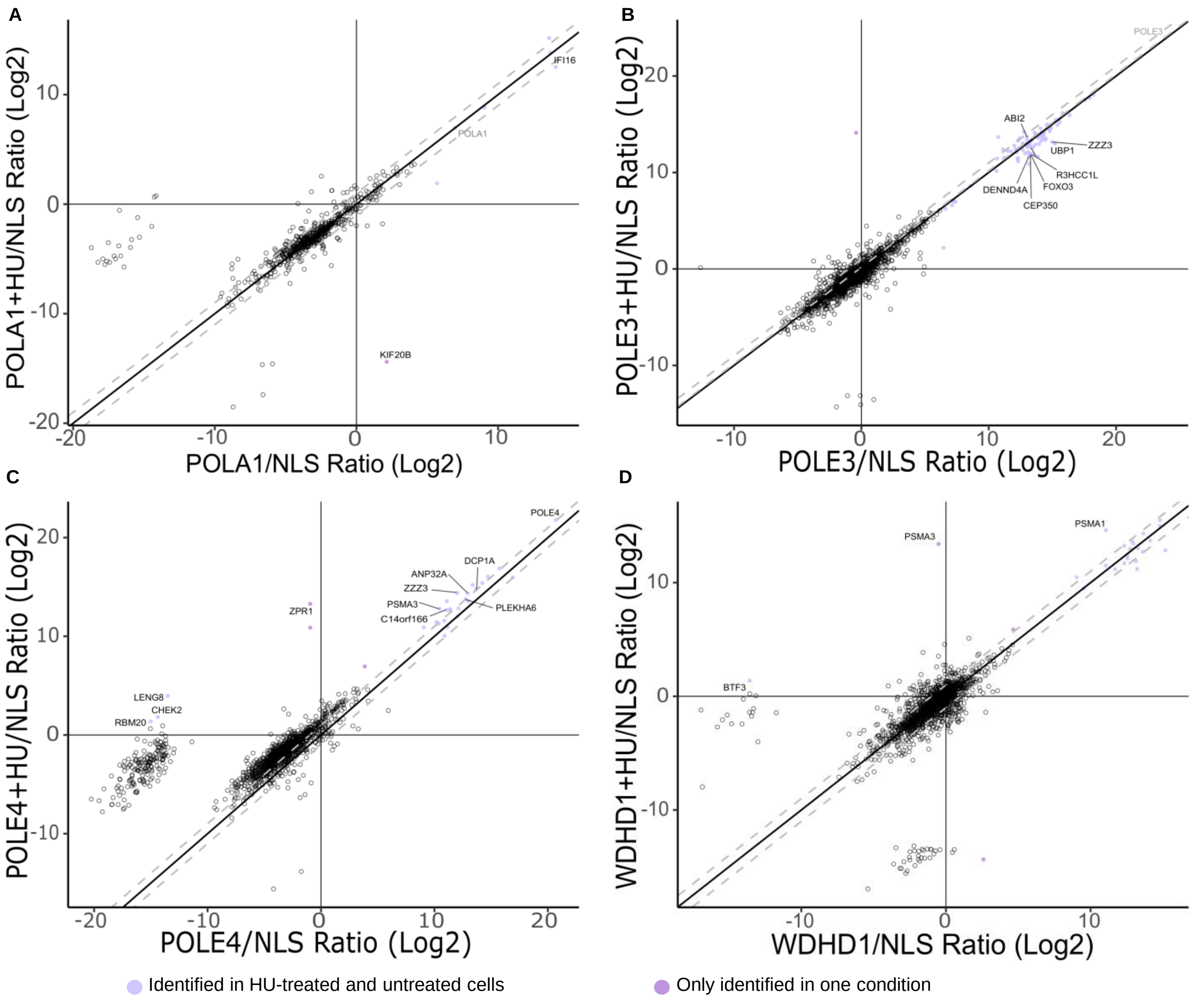
A
POLA1+HU/NLS Ratio (Log2)
POLA1/NLS Ratio (Log2)
IFI16
POLA1
KIF20B
10
0
-10
-20
B
POLE3+HU/NLS Ratio (Log2)
POLE3/NLS Ratio (Log2)
POLE3
ABI2
ZZZ3
UBP1
R3HCC1L
FOXO3
DENND4A
CEP350
20
C
POLE4+HU/NLS Ratio (Log2)
POLE4/NLS Ratio (Log2)
POLE4
DCP1A
ANP32A
ZZZ3
PSMA3
PLEKHA6
C14orf166
ZPR1
LENG8
CHEK2
RBM20
D
WDHD1+HU/NLS Ratio (Log2)
WDHD1/NLS Ratio (Log2)
PSMA1
PSMA3
BTF3
Identified in HU-treated and untreated cells
Only identified in one condition

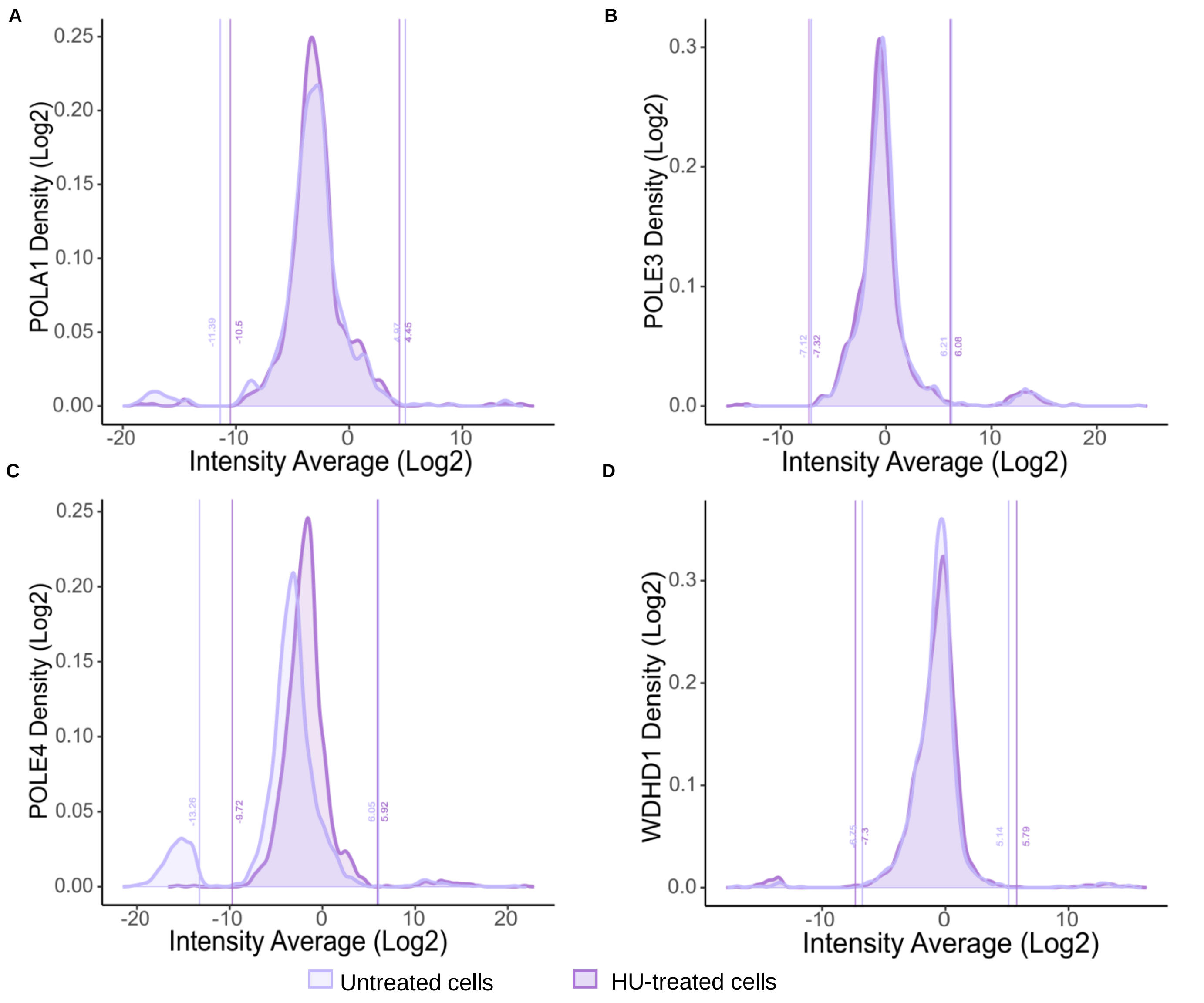
A
POLA1 Density (Log2)
Intensity Average (Log2)
-11.39
-10.5
4.97
4.45
B
POLE3 Density (Log2)
Intensity Average (Log2)
-7.12
-7.32
6.21
6.08
C
POLE4 Density (Log2)
Intensity Average (Log2)
-13.26
-9.72
6.05
5.92
D
WDHD1 Density (Log2)
Intensity Average (Log2)
-6.75
-7.3
5.14
5.79
Untreated cells
HU-treated cells

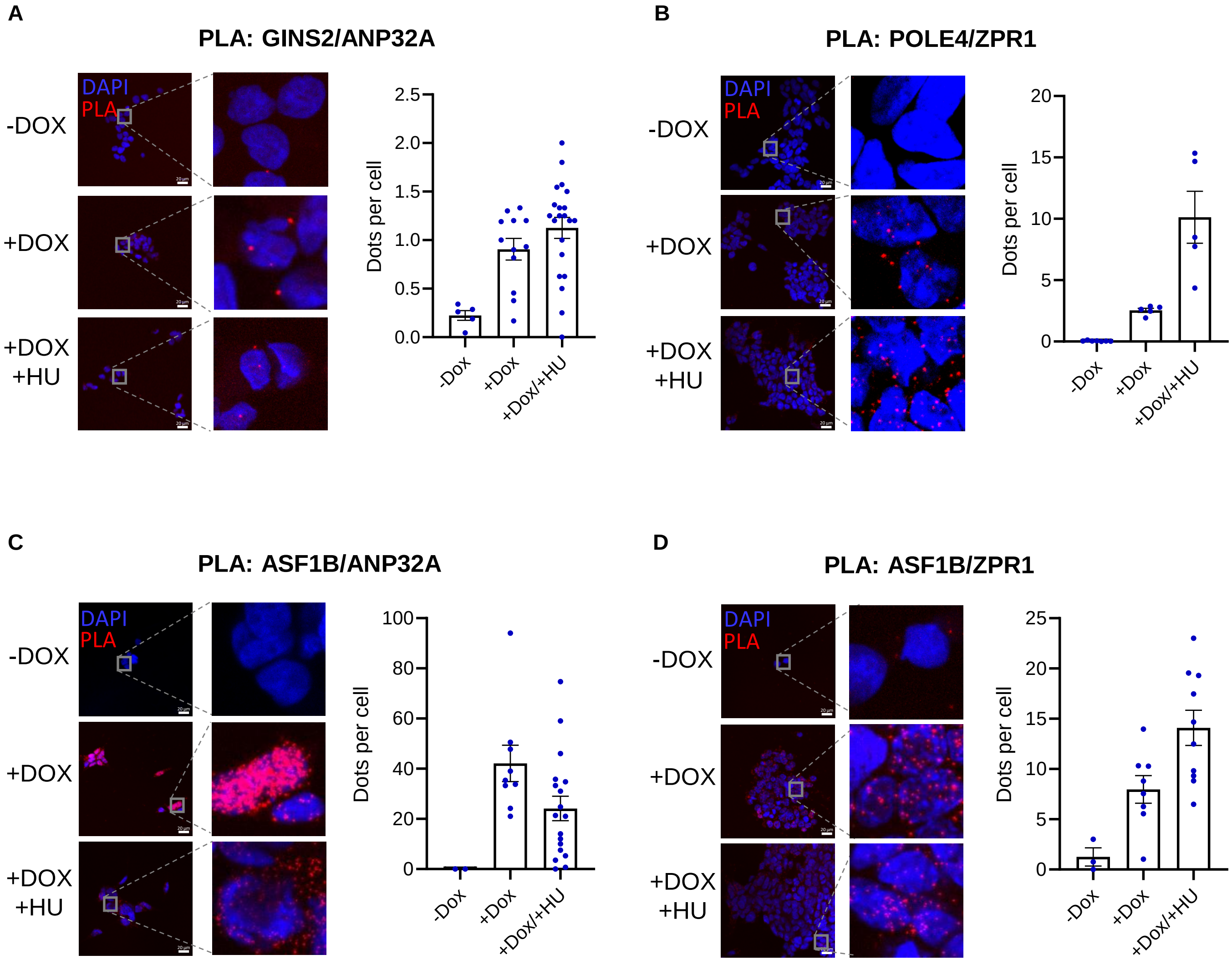

A
PLA: GINS2/ANP32A
-DOX
+DOX
+DOX +HU
DAPI
PLA
Dots per cell
0.0
0.5
1.0
1.5
2.0
2.5
-Dox
+Dox
+Dox/+HU
B
PLA: POLE4/ZPR1
0
5
10
15
20
C
PLA: ASF1B/ANP32A
0
20
40
60
80
100
D
PLA: ASF1B/ZPR1
25

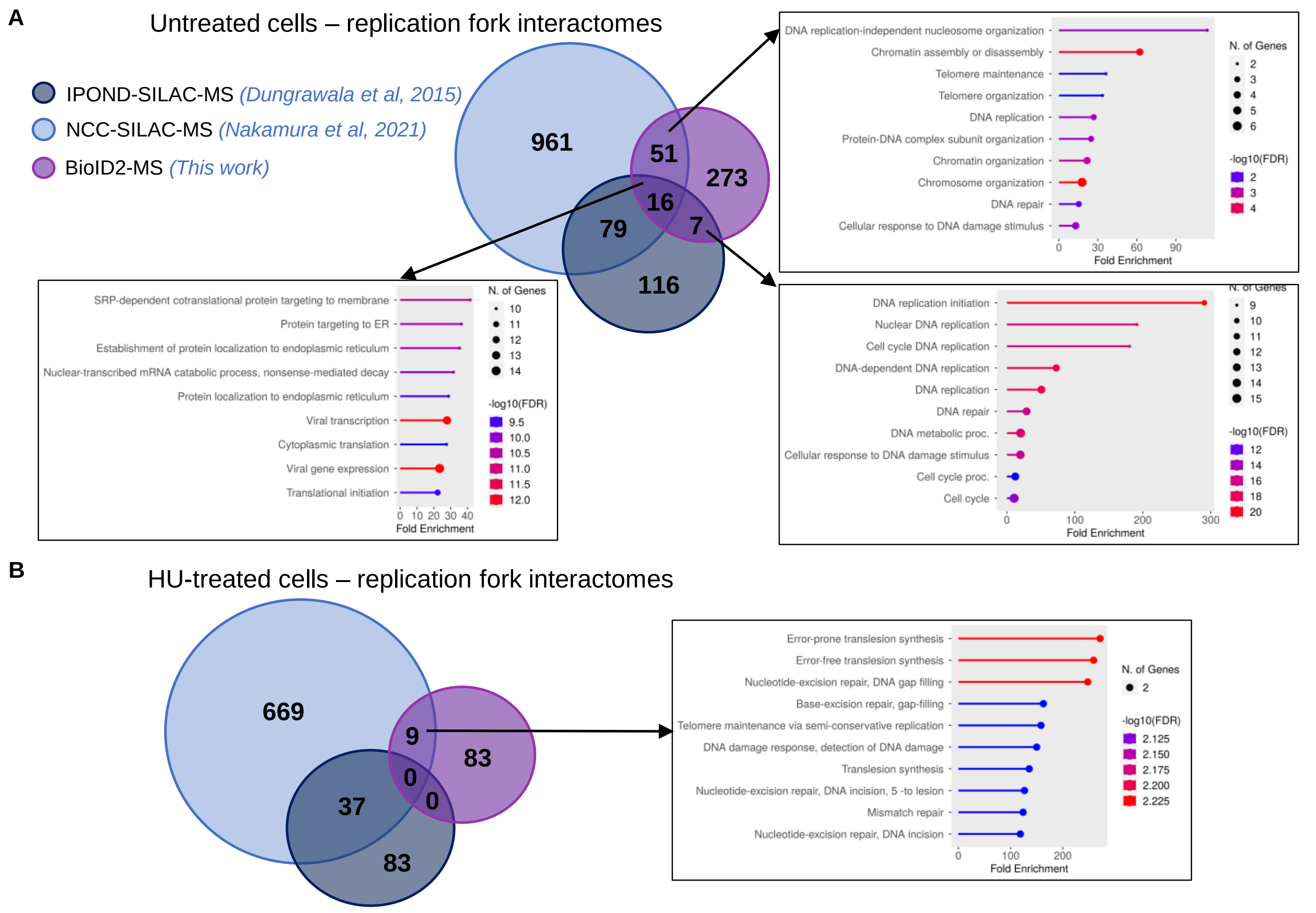
A
Untreated cells – replication fork interactomes
IPOND-SILAC-MS (Dungrawala et al, 2015)
NCC-SILAC-MS (Nakamura et al, 2021)
BioID2-MS (This work)
961
51
273
16
79
7
116
DNA replication-independent nucleosome organization
Chromatin assembly or disassembly
Telomere maintenance
Telomere organization
DNA replication
Protein-DNA complex subunit organization
Chromatin organization
Chromosome organization
DNA repair
Cellular response to DNA damage stimulus
0 30 60 90
Fold Enrichment
N. of Genes
2 3 4 5 6
-log10(FDR)
2 3 4
SRP-dependent cotranslational protein targeting to membrane
Protein targeting to ER
Establishment of protein localization to endoplasmic reticulum
Nuclear-transcribed mRNA catabolic process, nonsense-mediated decay
Protein localization to endoplasmic reticulum
Viral transcription
Cytoplasmic translation
Viral gene expression
Translational initiation
0 10 20 30 40
Fold Enrichment
N. of Genes
10 11 12 13 14
-log10(FDR)
9.5 10.0 10.5 11.0 11.5 12.0
DNA replication initiation
Nuclear DNA replication
Cell cycle DNA replication
DNA-dependent DNA replication
DNA replication
DNA repair
DNA metabolic proc.
Cellular response to DNA damage stimulus
Cell cycle proc.
Cell cycle
0 100 200 300
Fold Enrichment
N. of Genes
9 10 11 12 13 14 15
-log10(FDR)
12 14 16 18 20
B
HU-treated cells – replication fork interactomes
669
9
83
0
37
0
83
Error-prone translesion synthesis
Error-free translesion synthesis
Nucleotide-excision repair, DNA gap filling
Base-excision repair, gap-filling
Telomere maintenance via semi-conservative replication
DNA damage response, detection of DNA damage
Translesion synthesis
Nucleotide-excision repair, DNA incision, 5 -to lesion
Mismatch repair
Nucleotide-excision repair, DNA incision
0 100 200
Fold Enrichment
N. of Genes
2
-log10(FDR)
2.125 2.150 2.175 2.200 2.225
